# Magnetic targeting of adoptively transferred tumour-specific nanoparticle-loaded CD8^+^ T cells does not improve their tumour infiltration in a mouse model of cancer but promotes the retention of these cells in tumour-draining lymph nodes

**DOI:** 10.1186/s12951-019-0520-0

**Published:** 2019-08-06

**Authors:** Laura Sanz-Ortega, Yadileiny Portilla, Sonia Pérez-Yagüe, Domingo F. Barber

**Affiliations:** Department of Immunology and Oncology, and NanoBiomedicine Initiative, Centro Nacional de Biotecnología (CNB)-CSIC, Darwin 3, Cantoblanco, 28049 Madrid, Spain

**Keywords:** Cell-based therapy, Effector T cell, Magnetic nanoparticle, Magnetic retention, Cancer immunotherapy

## Abstract

**Background:**

Adoptive T cell-transfer (ATC) therapy is a highly promising cancer-treatment approach. However, in vivo-administered T cells tend to disperse, with only a small proportion reaching the tumour. To remedy this, magnetic targeting of T cells has been recently explored. Magnetic nanoparticles (MNPs) functionalised with antibodies were attached to effector T cells and magnetically recruited to tumour sites under MRI guidance. In this study, we investigated whether 3-aminopropyl-triethoxysilane (APS)-coated MNPs directly attached to CD8^+^ T cell membranes could also magnetically target and accumulate tumour-specific CD8^+^ T cells in solid tumours using an external magnetic field (EMF). As it has been shown that T cells associated with APS-coated MNPs are retained in lymph nodes (LNs), and tumour-draining LNs are the most common sites of solid-tumour metastases, we further evaluated whether magnetic targeting of APS-MNP-loaded CD8^+^ T cells could cause them to accumulate in tumour-draining LNs.

**Results:**

First, we show that antigen-specific CD8^+^ T cells preserve their antitumor activity in vitro when associated with APS-MNPs. Next, we demonstrate that the application of a magnetic field enhanced the retention of APS-MNP-loaded OT-I CD8^+^ T cells under flow conditions in vitro. Using a syngeneic mouse model, we found similar numbers of APS-MNP-loaded OT-I CD8^+^ T cells and OT-I CD8^+^ T cells infiltrating the tumour 14 days after cell transfer. However, when a magnet was placed near the tumour during the transfer of tumour-specific APS-MNP-loaded CD8^+^ T cells to improve tumour infiltration, a reduced percentage of tumour-specific T cells was found infiltrating the tumour 14 days after cell transfer, which was reflected in a smaller reduction in tumour size compared to tumour-specific CD8^+^ T cells transferred with or without MNPs in the absence of a magnetic field. Nonetheless, magnet placement near the tumour site during cell transfer induced infiltration of activated tumour-specific CD8^+^ T cells in tumour-draining LNs, which remained 14 days after cell transfer.

**Conclusions:**

The use of an EMF to improve targeting of tumour-specific T cells modified with APS-MNPs reduced the percentage of these cells infiltrating the tumour, but promoted the retention and the persistence of these cells in the tumour-draining LNs. 
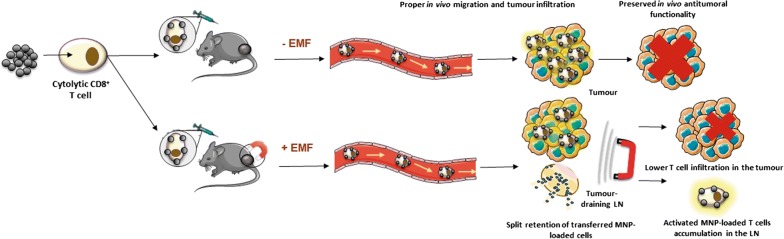

**Electronic supplementary material:**

The online version of this article (10.1186/s12951-019-0520-0) contains supplementary material, which is available to authorized users.

## Background

Cancer is one of the leading causes of death worldwide, and devising therapies that prevent tumour progression is a central goal. For decades, cancer therapy has mostly been limited to surgery, chemotherapy, and radiotherapy. However, the high relapse rates and numerous side effects of conventional approaches create a pressing need for novel treatments. One alternative that has recently re-emerged is immunotherapy, which seeks to slow or reverse tumour growth by modulating the immune response [1]. In manipulating the immune system to restore immunologic balance, immunotherapy can overcome immune-escape mechanisms and establish an effective antitumor response [2].

Of the different strategies used in cancer immunotherapy [3, 4], adoptive cell therapy (ACT) is one of the most promising. ACT isolates immune cells from a patient or donor, which are then modified, activated, expanded ex vivo under optimal conditions, and transferred back into the patient [5, 6], where they migrate to the tumour and mount an antitumor response. To do so, however, a sufficient quantity of cells must infiltrate the tumour, which involves dynamic and highly regulated processes. In several cancers, an optimal antitumor response correlates positively with increased infiltration of effector cells exhibiting antitumor activity, such as CD8^+^ T cells [7–9]. Therefore, ACT effectiveness depends on a number of factors, including the proliferation potential of transferred cells [10, 11], their persistence [12] or differentiation state [13], and their ability to migrate to and infiltrate the tumour [14, 15]. Another potential hurdle when applying ATC concerns the need to isolate and expand a large enough number of tumour-specific T cells for transfer [16].

ACT has achieved high regression rates in several cancer types, such as metastatic melanoma; however, lower rates have been reported in other cancers [17–19]. In many cases, only a small percentage of transferred cells infiltrate the tumour, as seen both in humans [20, 21] and mice [22, 23]. In addition, these cells may travel indiscriminately to multiple organs [24], potentially causing disease. Some reasons for low traffic to the tumour site include the mismatch between chemokine receptors and the chemokine pool in the tumour environment [25–27], a decrease in adhesion molecules, and aberrant vasculature, which can promote irregular blood flow and therefore inefficient immune-cell traffic in the tumour [28] and the tumour endothelium, the latter potentially acting as a barrier against effector-cell infiltration [29]. Therefore, a formidable challenge in ACT is to design strategies that promote specific cell infiltration, accumulation, and survival in the tumour microenvironment. Many of these new strategies are focused on certain chemokine receptors, integrins, endothelial factors, or homing peptides. These, however, require specific mechanisms for different types of tumours, and the design of more versatile approaches would simplify use of ACT across a wide range of tumour types.

Approaches based on nanoparticles could provide such versatility. In particular, magnetic approaches have successfully targeted tumours using magnetic nanoparticles (MNPs). This strategy enables specific release of drugs and biomolecules at the tumour site, which increases the concentration of these molecules at the site while reducing their concentration in blood, leading to lower systemic toxicity. This indicates that MNPs can be targeted to a desired area using an external magnetic field (EMF) [30]. Encouraged by these and other results, several subsequent studies have explored the use of magnetic targeting to direct cells to specific locations. MNP-loaded stem or mesenchymal cells, macrophages, and DCs have been tested in tissue-regeneration therapies and for autoimmune disorders [31–35]. Lymphoid cells have characteristically high motility and are continuously in circulation, responding to chemokine gradients, which are crucial for directing their movement to specific sites. Very few studies have investigated whether MNPs or EMFs could affect the migration and functionality of immune cells [36, 37]. Recently, our group explored MNP-mediated magnetic targeting of T cells, showing that MNPs attached to the primary T cell surface did not affect basic cell functions and that by using a magnet, T cells loaded with APS-MNPs could be retained in lymph nodes (LNs) [38]. More recently, magnetic nanoclusters modified with an anti-PD-1 antibody were bound to effector T cells to promote the MRI-guided magnetic recruitment of both effector T cells and PD-1 antibody in solid tumours [39]. These results prompted us to evaluate whether APS-coated MNPs attached to CD8^+^ T cell membranes could also magnetically target and accumulate tumour-specific CD8^+^ T cells in a solid tumour and in its draining LNs with the use of a magnet; though less sophisticated than antibody-modified MNPs and magnetic recruitment with MRI guidance, this approach is easier to undertake. Since we previously showed that APS-MNP-loaded T cells had greater retention in LNs, and given that one of the most common sites of solid tumour metastases are tumour-draining LNs, we also explored whether magnetic retention could also cause these T cells to remain in the tumour-draining LN due to the proximity between both tissue structures [40].

The most widely used tumour models to study specific antitumor T cell responses are syngeneic tumours expressing a specific ovoalbumin (OVA)-derived antigenic peptide presented by H-2 Kb in combination with adoptive transfer of CD8^+^ T cells from OT-I mice expressing a transgenic TCR that specifically recognises the combination of OVA peptide and H-2 Kb [41]. Use of this tumour model, in which rejection depends mainly on these tumour-specific CD8^+^ T cells [42–44], allowed us to evaluate whether MNPs and EMFs could increase retention of these CD8^+^ T cells in the tumour and, if so, whether this led to improved treatment efficacy.

First, we performed an in vitro analysis of different functional aspects of CD8^+^ T cells associated with MNPs, such as their ability to conjugate with and lyse target cells, as well as their capacity to degranulate and produce IFN-γ. We then tested the retention efficacy of antigen-specific CD8^+^ T cells associated with MNPs in vivo using a tumour model where the tumour cells express the antigen recognised by transferred CD8^+^ T cells. Placing a magnet near the tumours during transfer of MNP-loaded CD8^+^ T cells did not increase tumour infiltration by these cells or decrease tumour volume compared to non-EMF-exposed tumours. However, application of an EMF close to the tumour when MNP-loaded CD8^+^ T cells were transferred resulted in a higher percentage of more activated tumour-infiltrating CD8^+^ T cells and greater accumulation of CD8^+^ T cells in the tumour-draining LN.

## Results

### Synthesis and physico-chemical characterisation of MNPs and analysis of their interaction with antitumour effector T cells

We synthesised MNPs with an iron oxide core of 12.5 nm, which were subsequently coated, obtaining negatively charged (dimercaptosuccinic acid (DMSA)-MNPs), positively charged (3-aminopropyl-triethoxysilane (APS)-MNPs), and non-charged MNPs (DEXT-MNPs). Then, the hydrodynamic size and the Z-potential of these MNPs were analysed by dynamic light scattering (DLS), the presence of coatings was verified by infrared spectrophotometry, and organic composition was examined by thermogravimetric analysis. A summary of the physico-chemical charasteristics of the MNPs used in this study appears in Table 1 (for further chemical and physical characterisation, see Sanz-Ortega et al. [38]).

**Table 1:** Characterisation of DMSA-MNPs, APS-MNPs, and DEXT-MNPs

Name	Coating	Hydrodynamic diameter (nm)	Z-potential (mV)	Organic composition (%)
DMSA-MNP	Dimercaptosuccinic acid	83	− 34	10
APS-MNP	3-Aminopropyl-triethoxysilane	82	+ 38	10
DEXT-MNP	Dextran 6 kDa	119	− 2	38

The antitumour effector T cells used for this study were major histocompatibility complex (MHC) class I-restricted, ovalbumin-specific CD8^+^ T cells from OT-I TCR transgenic (OT-I) mice. OT-I mice were routinely phenotyped by FACS to determine the expression level of Vα2 (α chain of the OT-I TCR) on the surface of CD8^+^ T cells within the CD90.2^+^ population of mature T cells (Additional file 1: Fig. S1). Only OT-I mice having high Vα2 expression were used. OT-I CD8^+^ T cells were obtained from single-cell suspensions prepared from the homogenised spleens and LNs of these transgenic mice. Then, transgenic CD8^+^ T cells were specifically activated and expanded in vitro by adding the OVA-specific peptide (OVA_257–264_) to the cell suspension for 2 days and subsequently adding IL-2 (Additional file 1: Fig. S2A, B) for 3 days in accordance with the protocols indicated in the materials and methods section. In addition, we verified the lack of expansion in the absence of the specific stimuli (Additional file 1: Fig. S2C).

**Fig. 1:**
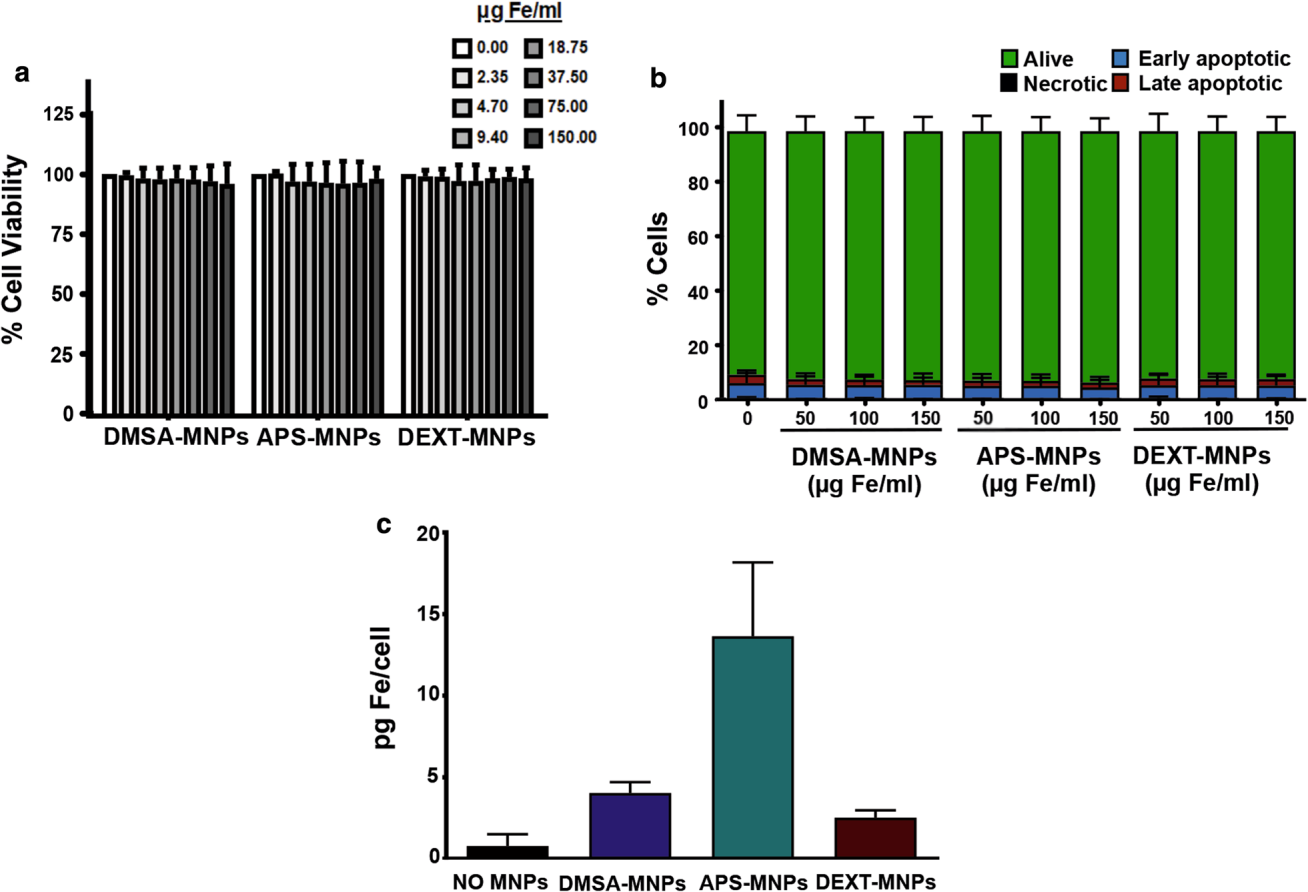
Evaluation of MNP toxicity and uptake by OT-I CD8^+^ T cells. **a** Cell viability of OT-I CD8^+^ T cells after treatment with MNPs, measured using the Alamar Blue fluorometric test. **b** Analysis by flow cytometry of the percentage of apoptotic or necrotic OT-I CD8^+^ T cells after incubation with MNPs by Annexin V/PI staining. **c** Quantification by ICP-OES of the iron associated with the OT-I CD8^+^ T cells after incubation with the different MNPs. Mean values ± SD are shown for three different experiments, *p < 0.05, **p < 0.01, ***p < 0.001

To assess the effects of MNP on OT-I CD8^+^ T cells, we first analysed MNP toxicity and uptake using different assays. Toxicity was assessed by Alamar Blue assay and by annexin V-FITC/PI flow cytometry. Both analyses revealed that none of the MNPs tested were toxic to these cells, finding no changes in the proportion of apoptotic or necrotic cells at the different dose levels used (Fig. 1a, b).

The amount of MNPs associated with OT-I CD8^+^ T cells was also evaluated. The results obtained after a 2-h incubation of these cells with the MNPs revealed that APS-MNPs gave rise to a higher iron concentration in the cell samples analysed (13.7 ± 4.5 pg Fe/cell using APS-MNPs vs 4.0 ± 0.7 and 2.5 ± 0.5 pg Fe/cell with DMSA-MNPs and DEXT-MNPs, respectively) (Fig. 1c).

**Fig. 2:**
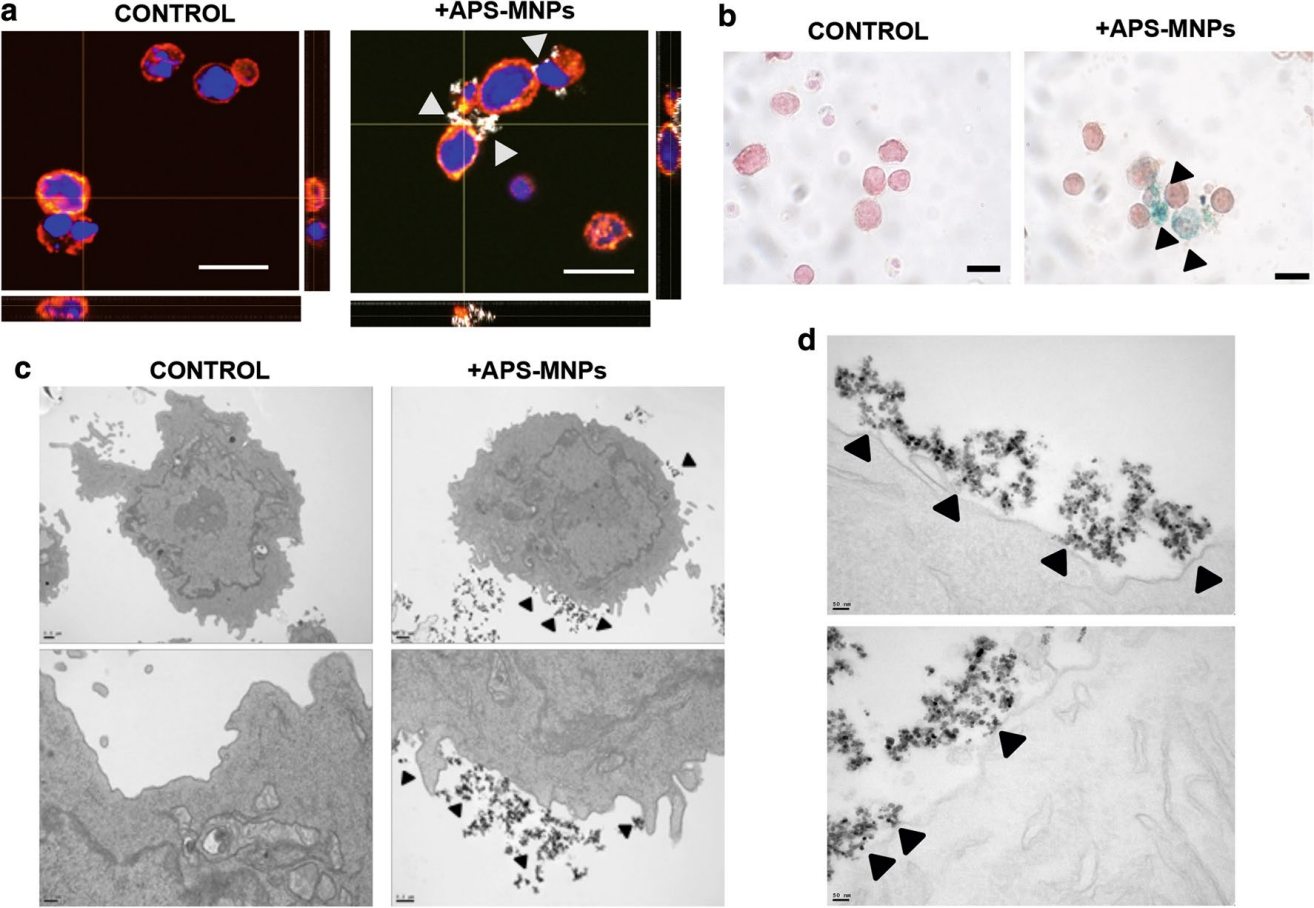
APS-MNP subcellular localisation in OT-I CD8^+^ T cells. **a** Representative images acquired using confocal microscopy of the OT-I CD8^+^ T cells after treatment with APS-MNPs (cell membrane (red), MNPs (grey), and core (blue)) (scale = 10 μm). The orthogonal projections were composed using the ImageJ software. **b** Prussian blue staining and neutral red counterstain of OT-I CD8^+^ T cells after association with the MNPs (scale = 10 μm). **c** Representative images obtained by TEM of OT-I CD8^+^ T cells after treatment with APS-MNPs. The upper panels offer an overall view of the cell, while the lower panels show cellular regions in greater detail to better illustrate the interactions between APS-MNPs and the cell membrane. **d** TEM images at greater magnifications showing further interaction between the APS-MNPs and the cell surface. The arrows indicate the presence of APS-MNPs associated with the cells

We next studied the subcellular location of APS-MNPs in OT-I CD8^+^ T cells using several microscopy techniques. After incubating these cells for 2 h with 150 µg Fe/ml of the previously mentioned MNPs, the cells were washed several times to eliminate excess MNPs and analysed either by confocal microscopy or bright field microscopy after Prussian blue staining. The resulting images showed that APS-MNPs were associated with the cell membrane (Fig. 2a, b). An analysis at higher resolution carried out through transmission electron microscopy further confirmed the close interaction between APS-MNPs and the cell membrane of OT-I CD8^+^ T cells (Fig. 2c, d).

### MNPs did not alter the OT-I CD8^+^ T cell surface phenotype or its main effector functions

**Fig. 3:**
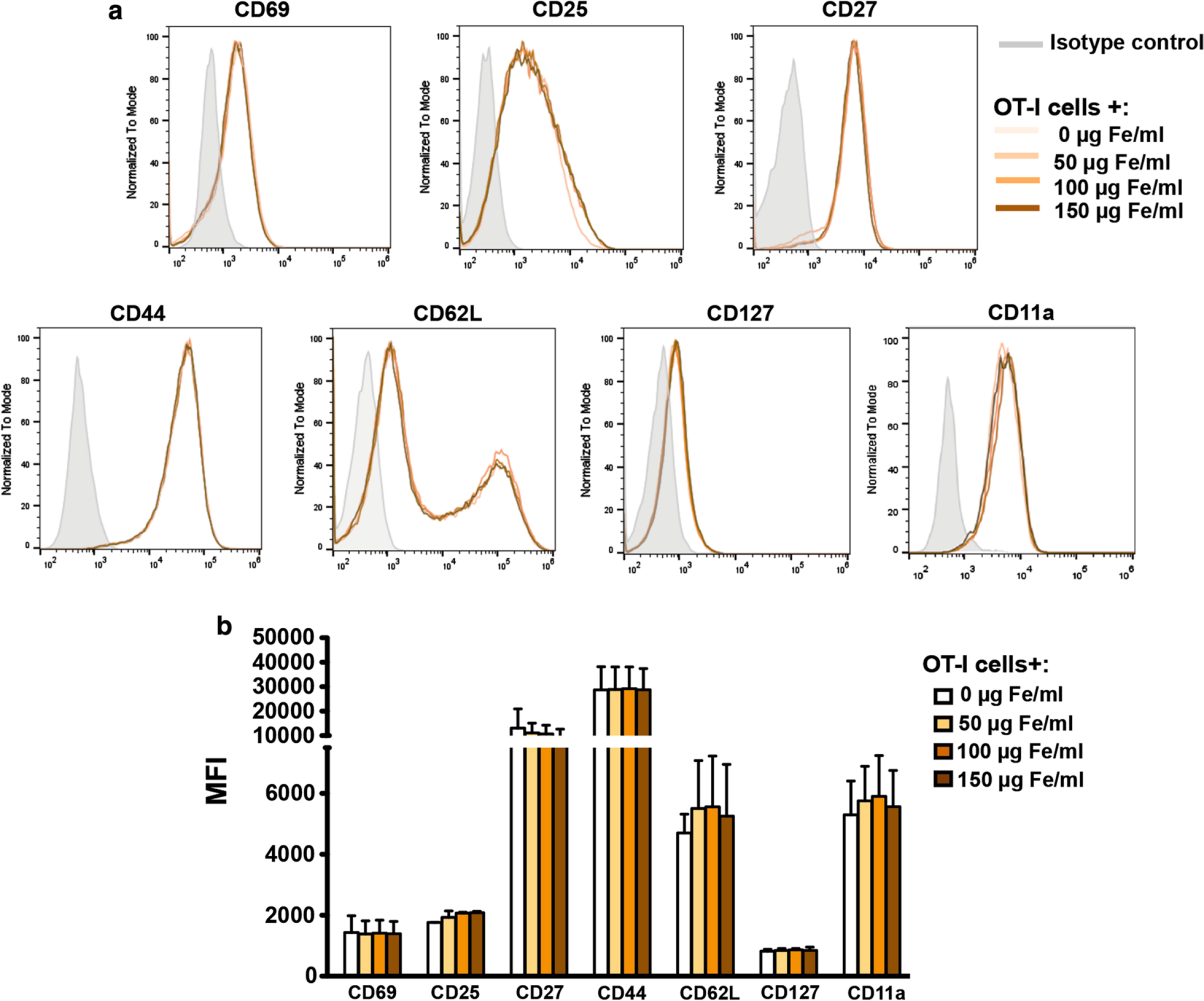
Phenotypic analysis of OT-I CD8^+^ T cells after association with APS-MNPs. **a** Representative histograms and **b** quantification (MFI, mean fluorescence intensity) of the expression of relevant cell-surface markers in OT-I CD8^+^ T cells, after incubation with APS-MNPs. Mean values ± SD are shown for three different experiments, *p < 0.05, **p < 0.01, ***p < 0.001

To check whether APS-MNP, in association with the OT-I CD8^+^ cell membrane, induced changes in the cell-surface phenotype, using flow cytometry we first analysed the expression levels of several T cell surface markers in OT-I CD8^+^ T cells after incubating these cells with APS-MNPs. Early and late activation markers such as CD69, CD27, CD44, and CD25 as well as the adhesion and memory molecules CD11a, CD62L, and CD127 were analysed. As seen in Fig. 3a, b, no significant differences were observed in the expression of the different markers analysed.

We next evaluated the influence of MNP over main effector functions of OT-I CD8^+^ T cells such as the induction of cytotoxicity in infected or transformed cells as well as the production of a range of cytokines like IFN-γ.

**Fig. 4:**
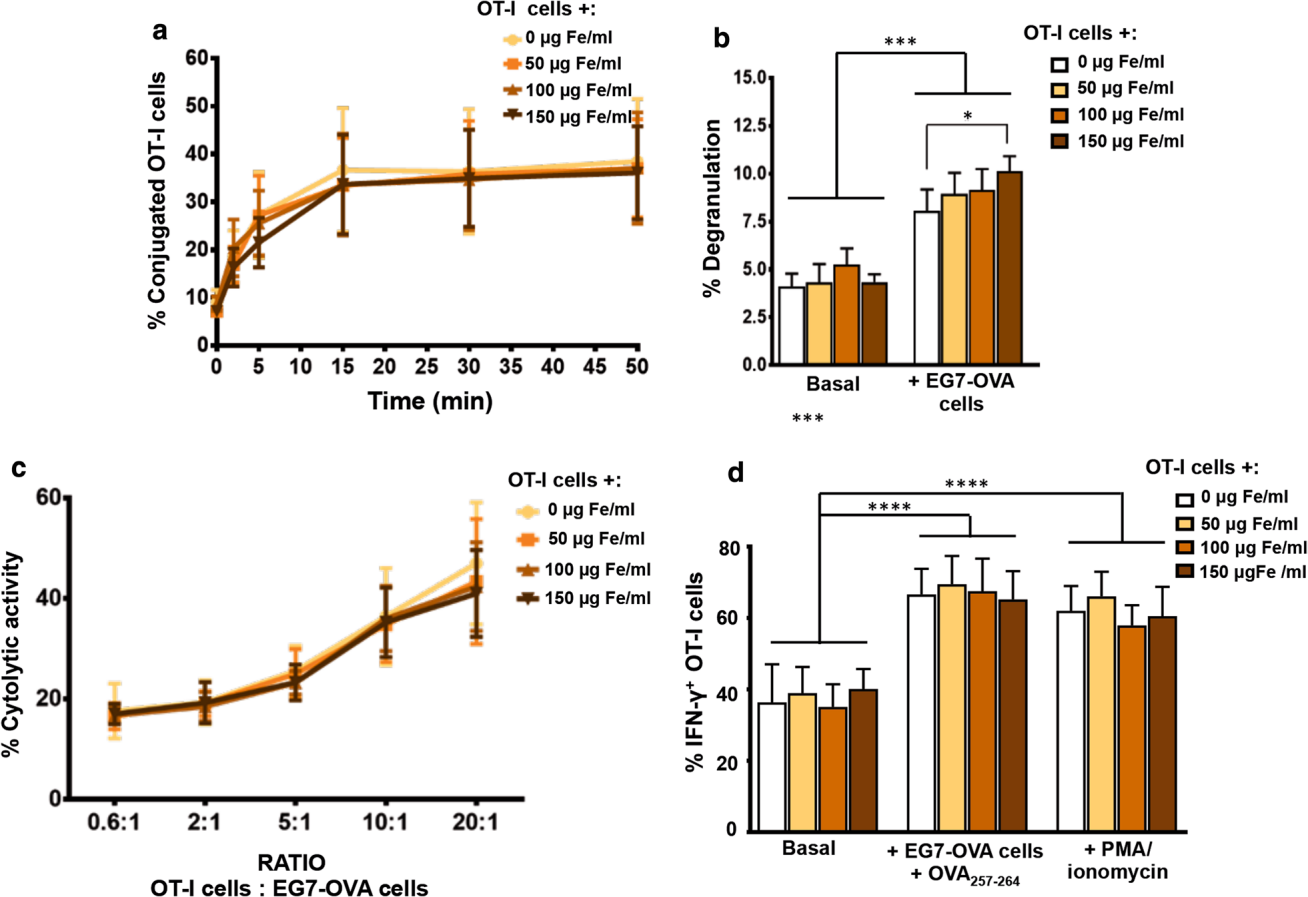
Functional assessment of OT-I CD8^+^ T cells after association with APS-MNPs. **a** Conjugation kinetics of OT-I CD8^+^ T cells after treatment with APS-MNPs, with the EG7-OVA cell line, co-incubated at a 1: 1 ratio. **b** Degranulation capacity of OT-I CD8^+^ T cells after being treated with different concentrations of APS-MNPs and co-incubated with the EG7-OVA cell line. **c** Cytolytic activity of OT-I CD8^+^ T cells after being treated with different concentrations of APS-MNPs, against EG7-OVA cell line, co-incubated at different ratios for 5 h. **d** Quantification of the percentage of OT-I CD8^+^ T cell IFN-γ^+^ after exposure to a variety of stimuli. Mean values ± SD are shown for three or four different experiments, *p < 0.05, **p < 0.01, ***p < 0.001, ****p < 0.0001

For a CD8^+^ T cell to exert its cytolytic activity against a target cell, there must first be physical interaction or conjugation between them [45]. We carried out conjugation experiments between OT-I CD8^+^ T cells and the EG7-OVA cell line, the latter expressing the OVA peptide presented by H-2 Kb on its surface, which is specifically recognised by OT-I CD8^+^ T cells. No significant differences in the conjugation kinetics of EG7-OVA cells and OT-I CD8^+^ T cells were detected when APS-MNPs were used and when associated with increasing APS-MNP concentrations (9.1 ± 5.2% vs 7.2 ± 2.3% of conjugation in the absence of APS-MNPs or in the presence of 150 µg Fe/ml of APS-MNPs, respectively, at 1 min, and 38.4 ± 13.2% vs 36.1 ± 11.3% at 45 min) (Fig. 4a).

After conjugation with target cells, CD8 ^+^ T cells are activated, which triggers cytolytic granule polarisation and degranulation toward target cells [46]. For this reason, we measured the post-activation degranulation capacity of APS-MNP-loaded and -free OT-I CD8^+^ T cells by analysing the presence of the CD107a marker on the membrane, which is increased in the cytolytic cells once they have been activated and the lytic granules have been released, this being an indirect measure of their cytolytic capacity [47]. The basal degranulation of these cells was not significantly altered due to the presence of the APS-MNPs, although a slight increase was seen in the degranulation of the OT-I CD8^+^ T cells after co-incubation with EG7-OVA cells when treated with the highest dose of APS-MNPs (8.0 ± 1.1% in the absence of MNPs vs 10.1 ± 0.8% in the presence of the highest dose of APS-MNPs after stimulation with EG7-OVA cells) (Fig. 4b).

The release of cytotoxic granules containing perforin and granzymes induces the lysis of target cells [46]. As has been shown, APS-MNPs do not seem to negatively affect OT-I CD8^+^ T cell conjugation and degranulation capacities, so the cytolytic capacity of OT-I in the presence of the APS-MNPs was further analysed. No significant difference was observed in the percentage of target cells lysed by the OT-I CD8^+^ T cells after their association with increasing concentrations of APS-MNPs in the different scenarios evaluated (17.6 ± 5.5% vs 17.0 ± 2.0% of lysis in the absence or presence of the highest dose of MNPs, respectively, at lower ratios, and 47.0 ± 12.2% vs 41.8 ± 8.7% at higher ratios) (Fig. 4c).

Another essential aspect in cytolytic CD8^+^ T cells is the ability to produce pro-inflammatory cytokines such as IFN-γ [48]. For this reason, the ability of APS-MNP-loaded OT-I CD8^+^ T cells to produce this cytokine after activation with both non-specific (PMA/ionomycin) and specific stimuli (EG7-OVA cells together with the OVA_257–264_ peptide) was assessed by flow cytometry. No significant differences were observed in the percentage of cells able to produce IFN-γ (Fig. 4d).

**Fig. 5:**
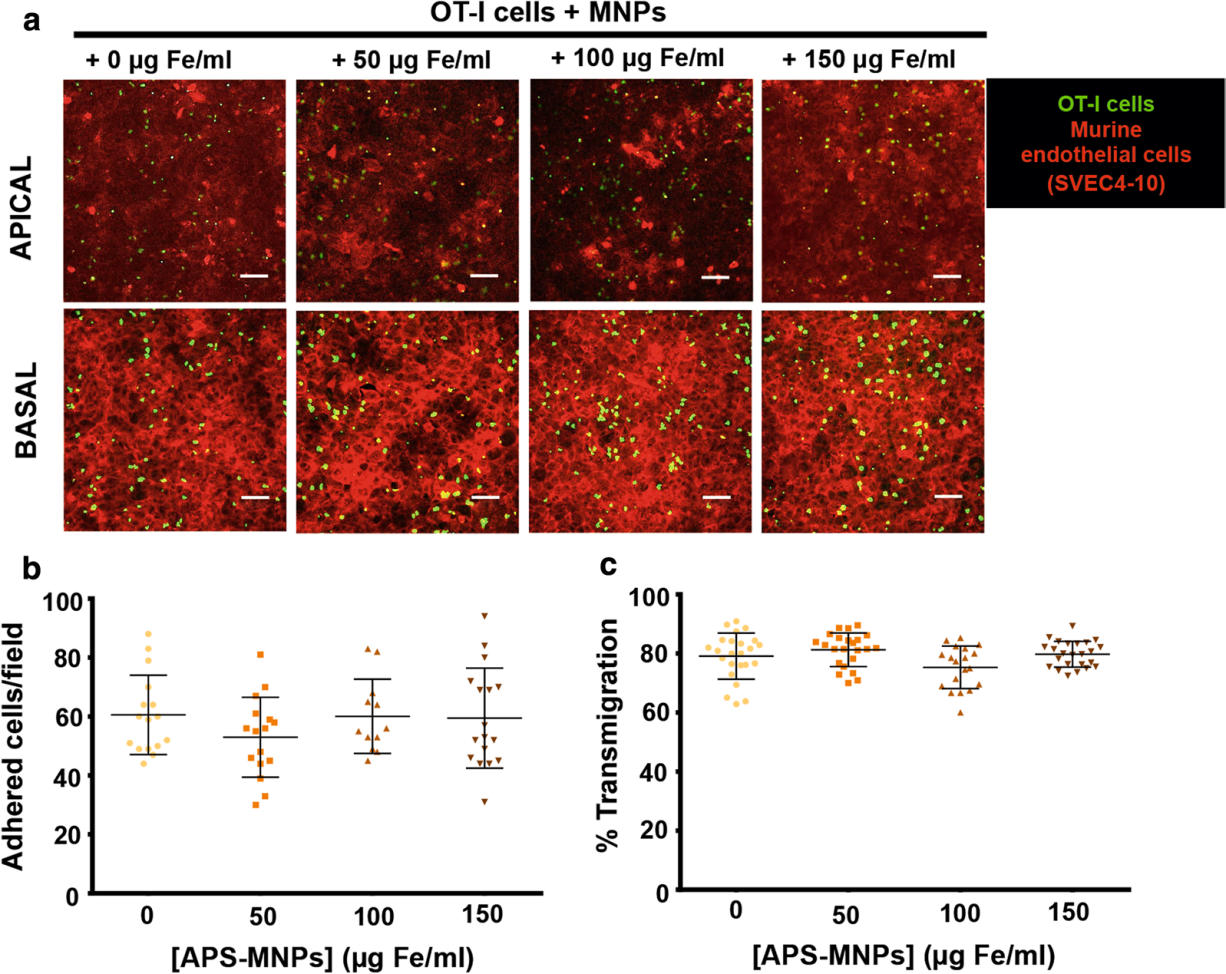
Adhesion and transmigration of APS-MNP-loaded OT-I CD8^+^ T cells across an endothelium. **a** Representative confocal microscopic images of OT-I CD8^+^ T cells, treated with APS-MNPs or not, migrating across a monolayer of murine endothelial cells (SVEC4-10 cell line). The upper panel represents the apical part of the monolayer, while the lower panel represents the basal part (OT-I CD8^+^ T cells (green), cytoskeleton (red)). Scale: 100 μm. Quantification of (**b**) adhesion and (**c**) transmigration of OT-I CD8^+^ T cells in the presence of increasing concentrations of APS-MNPs across murine endothelial cells. Mean values ± SD are shown for three different experiments, analysing at least 50 fields per condition, *p < 0.05, **p < 0.01, ***p < 0.001

Cells with antitumor activity are continuously circulating and can be recruited to the tumour by different processes related to adhesion to the endothelium and extravasation [49, 50]. Therefore, the capacity of adhesion to an endothelium and the transmigration capacity of OT-I CD8^+^ T cells across the edothelium were evaluated. No significant differences were observed in either the adhesion capacity (~ 60 adhered cells/field) (Fig. 5a, b) or in the ability to transmigrate (~ 75–80%) (Fig. 5a, c) of OT-I CD8^+^ T cells associated with increasing concentrations of APS-MNPs.

### Magnetic fields enhanced in vitro OT-I CD8^+^ T cell retention under flow conditions

**Fig. 6:**
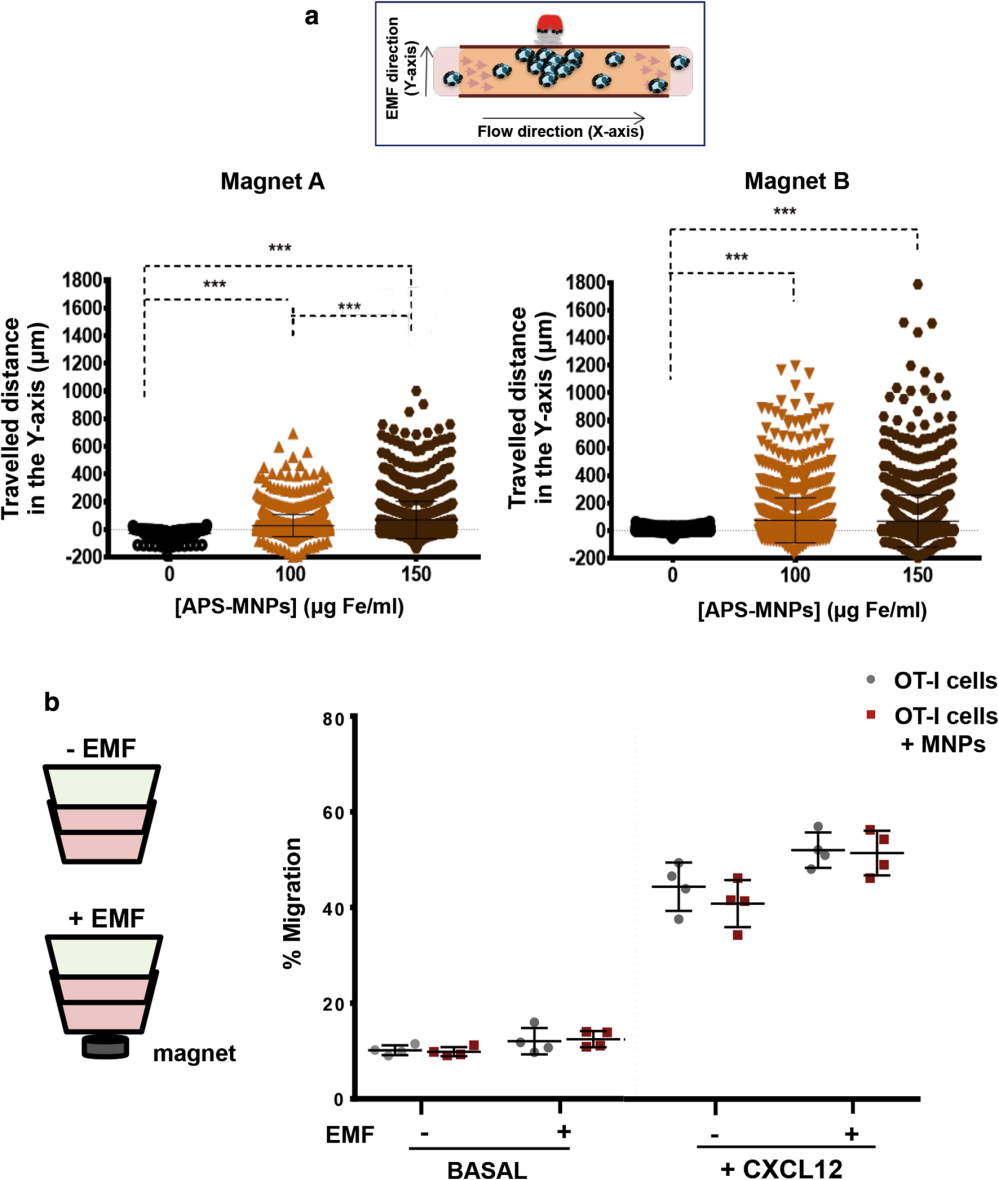
*In vitro* magnetic retention of APS-MNP-loaded OT-I CD8^+^ T cells and their chemotactic response. **a** Displacement of OT-I CD8^+^ T cells in the direction of the magnetic gradient (Y axis) after being treated with APS-MNPs or not and exposed to different EMFs. Cell displacement was quantified by analysing at least 100 cells per video using Imaris software. **b** Migratory capacity of OT-I CD8^+^ T cells after treatment with APS-MNPs in response to a specific chemotactic gradient and in the presence or absence of an EMF in the same direction. The results were normalised against a control well (in absence of a transwell assay). The results shown (mean ± SD) are representative of three or four independent experiments, *p < 0.05, **p < 0.01, ***p < 0.001

Magnetic retention of OT-I CD8^+^ T cells associated with APS-MNPs was evaluated in a dynamic flow system using magnets with different magnetic forces (magnets A and B, Table 2). This magnetic retention was greater with increases in the associated APS-MNPs (28 ± 80 μm with 100 μg Fe/ml vs 69 ± 134 μm with 150 μg Fe/ml using magnet A), as well as with increased magnetic gradient force (69 ± 134 μm with magnet A vs 90 ± 188 μm with magnet B, with the highest dose of MNPs in both cases) (Fig. 6a).

**Table 2:** Summary of the magnetic properties of the different magnets used

Magnet	Remanent magnetisation (Br)	Radius (mm)	Length (mm)
A	1.35	5	14
B	1.45	8	6

Because the chemotactic response guides the movement of the lymphoid cells through the different tissues to the regions where their activity is required, we evaluated the ability of OT-I CD8^+^ T cells both with and without cell surface associated APS-MNPs to migrate in response to an CXCL12 gradient. The results showed that the presence of APS-MNPs in the OT-I CD8^+^ T cells affected their migration in a slight but not significant way in response to a chemotactic gradient (44.4 ± 5.0 vs 40.9 ± 4.9% migration in the absence or presence of MNPs, respectively) (Fig. 6b). In addition, application of an EMF in the same direction as the chemotactic gradient appeared to produce an increase in the migration of APS-MNP-loaded OT-I CD8^+^ T cells (40.9 ± 4.9% vs 52.3 ± 4.7% of migration of the cells associated with APS-MNPs in the absence or presence of an EMF), though this increase was not significant (Fig. 6b).

### Though APS-MNP-loaded OT-I CD8^+^ T cells maintain their in vivo antitumour capacity, application of an EMF for magnetic targeting has an apparently negative effect

In order to evaluate the in vivo efficacy of the combined use of MNPs and EMFs in the transfer of antitumoral cells to treat cancer, we used a murine syngeneic tumour model, where the implanted EG7-OVA tumour cells express an OVA antigen presesented by H-2 Kb and which is specifically recognised by OT-I CD8^+^ T cells.

**Fig. 7:**
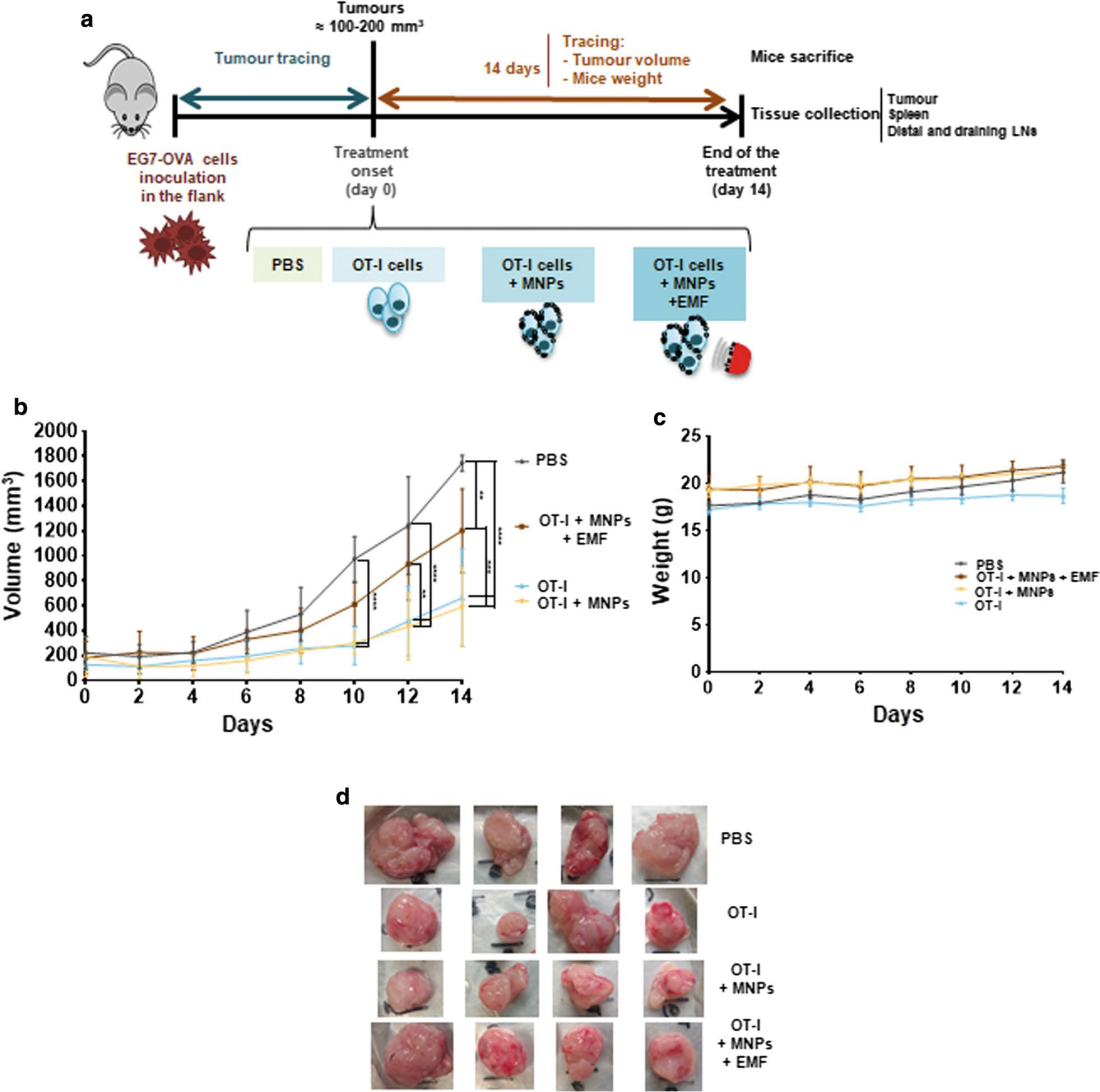
Evolution of tumour size and weight among mice after the different treatments. **a** Setting **b** Changes in tumour volume and **c** weight of the mice over the 14 days after the start of treatment among the different treatment groups. The results shown (mean ± SD) reflect 4 or 5 mice/group, *p < 0.05, **p < 0.01, ***p < 0.001, ****p < 0.0001. **d** Photographs of tumours classified by treatment group following post-sacrifice extraction at the end of the treatment

Once tumours were established by subcutaneous injection of EG7-OVA cells into the flanks of C57BL/6 mice, the different treatments were administered intravenously. Mice were randomised into 4 groups, receiving an inoculation of PBS as a control, OT-I CD8^+^ T cells, APS-MNP-loaded OT-I CD8^+^ T cells, or APS-MNP-loaded OT-I CD8^+^ T cells together with the application of an EMF over the tumour for approximately 90 min (Fig. 7a). The tumour size, as well as the weight of the different mice, was monitored for 2 weeks after treatment (Fig. 7b, c). Markedly significant differences in tumour size were observed as of day 10 post-treatment. The control group, which was treated with PBS, had the largest tumours (1744 ± 63 mm^3^), while the groups treated with OT-I CD8^+^ T cells without APS-MNPs or with APS-MNP-loaded OT-I CD8^+^ cells showed the lowest tumour growth (662 ± 391 mm^3^ and 590 ± 315 mm^3^, respectively). The group inoculated with APS-MNP-loaded OT-I CD8^+^ T cells that had been exposed to an EMF showed intermediate growth (1200 ± 337 mm^3^) (Fig. 7b). In addition, no differences in weight were observed between the different treatment groups, suggesting that the treatments did not induce any observable sign of toxicity (Fig. 7c).

### EMF-exposed tumours exhibited less infiltrated APS-MNP-loaded OT-I CD8^+^ T cells but had an activated phenotype

To understand why the exposition of an EMF appeared to reduce the ability of APS-MNP-loaded OT-I CD8^+^ T cells to control tumour growth, we analysed the different cell populations found in the tumour as well as different secondary lymphoid organs. After 14 days of follow-up (Fig. 7a–d), mice were sacrificed and different tissue structures, including the tumour as well as the tumour-draining and distal LNs and the spleen, were collected for further analysis.

**Fig. 8:**
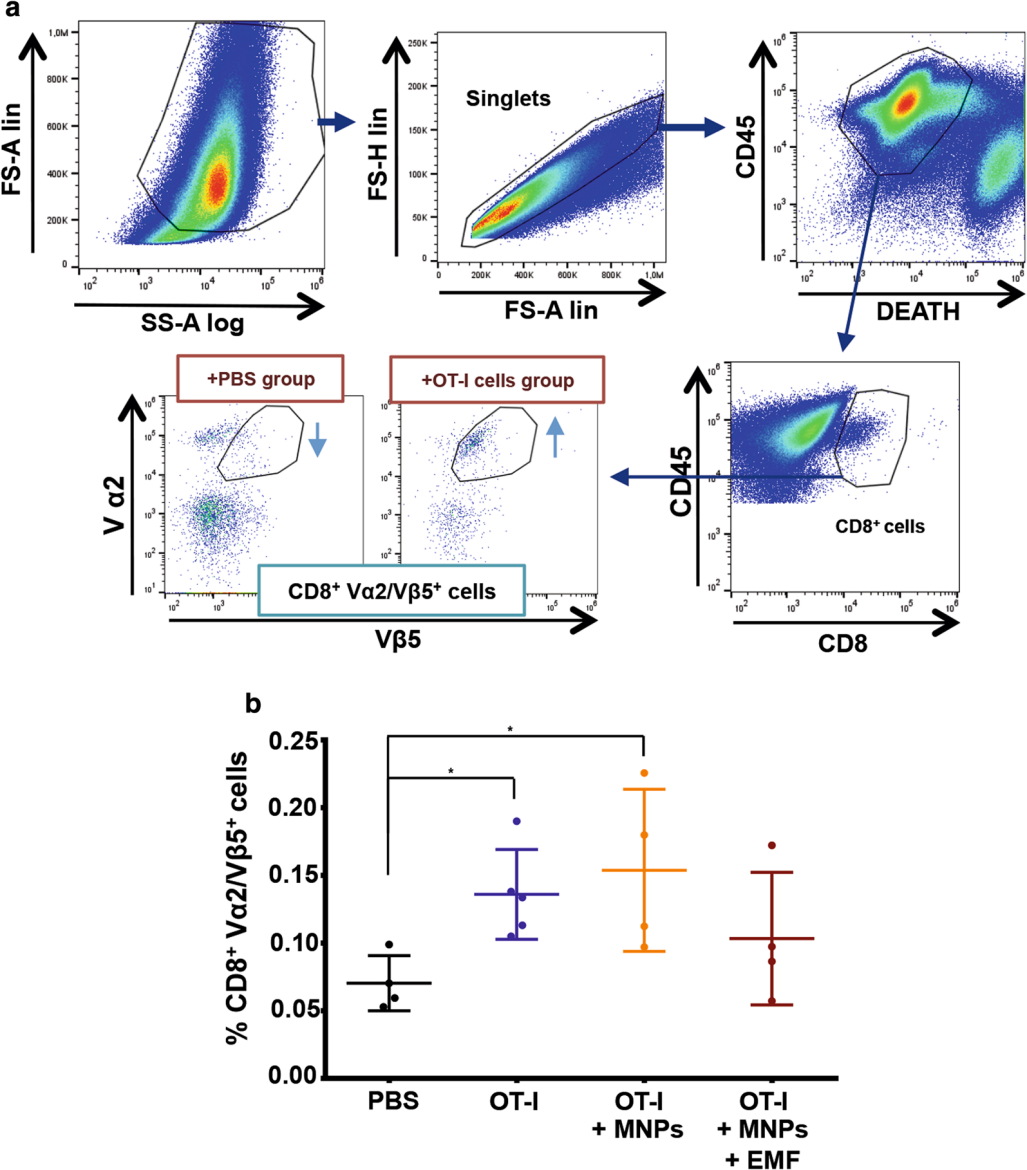
Infiltration of CD8^+^ Vα2/Vβ5^+^ T cells in the tumour in the different treatment groups. **a** Gating strategy followed to analyse the percentage of CD8^+^ Vα2/Vβ5^+^ T cells infiltrated in the tumour. **b** Percentage of CD8^+^ Vα2/Vβ5^+^ T cells with respect to the percentage of living CD45^+^ cells infiltrated in the tumour in the different treatment groups. The results shown (mean ± SD) correspond to 4 or 5 mice/group, *p < 0.05, **p < 0.01, ***p < 0.001

To analyse the phenotypic characteristics of the transferred OT-I CD8^+^ T cells that had infiltrated the tumour, we followed the gating strategy depicted in Fig. 8a. Briefly, after delimitating the cell population by morphology (FSlin vs SSlog), the population of living CD45^+^ cells within the singlets (FS-H vs FS-A) was selected. Subsequently, we selected those CD8^+^ cells that co-expressed Vα2 and Vβ5, which are specific for the transgenic TCR of the OT-I CD8^+^ T cells [51, 52] (Fig. 8a). The final percentage of infiltrating CD8^+^ Vα2/Vβ5^+^ T cells was expressed in relation to the percentage of living CD45^+^ cells (Fig. 8b). An analysis of the CD8^+^ Vα2/Vβ5^+^ T cell infiltration in the tumour revealed that the PBS group had the lowest percentage of infiltration (0.07 ± 0.03%), while the highest percentage of transferred cells was found in the groups inoculated with OT-I CD8^+^ T cells or APS-MNP-loaded OT-I CD8^+^ T cells but had not been exposed to an EMF (0.14 ± 0.03% and 0.14 ± 0.07%, respectively). The group treated with APS-MNP-loaded OT-I CD8^+^ T cells and exposed to EMF presented an intermediate value (0.11 ± 0.05%) (Fig. 8b).

**Fig. 9:**
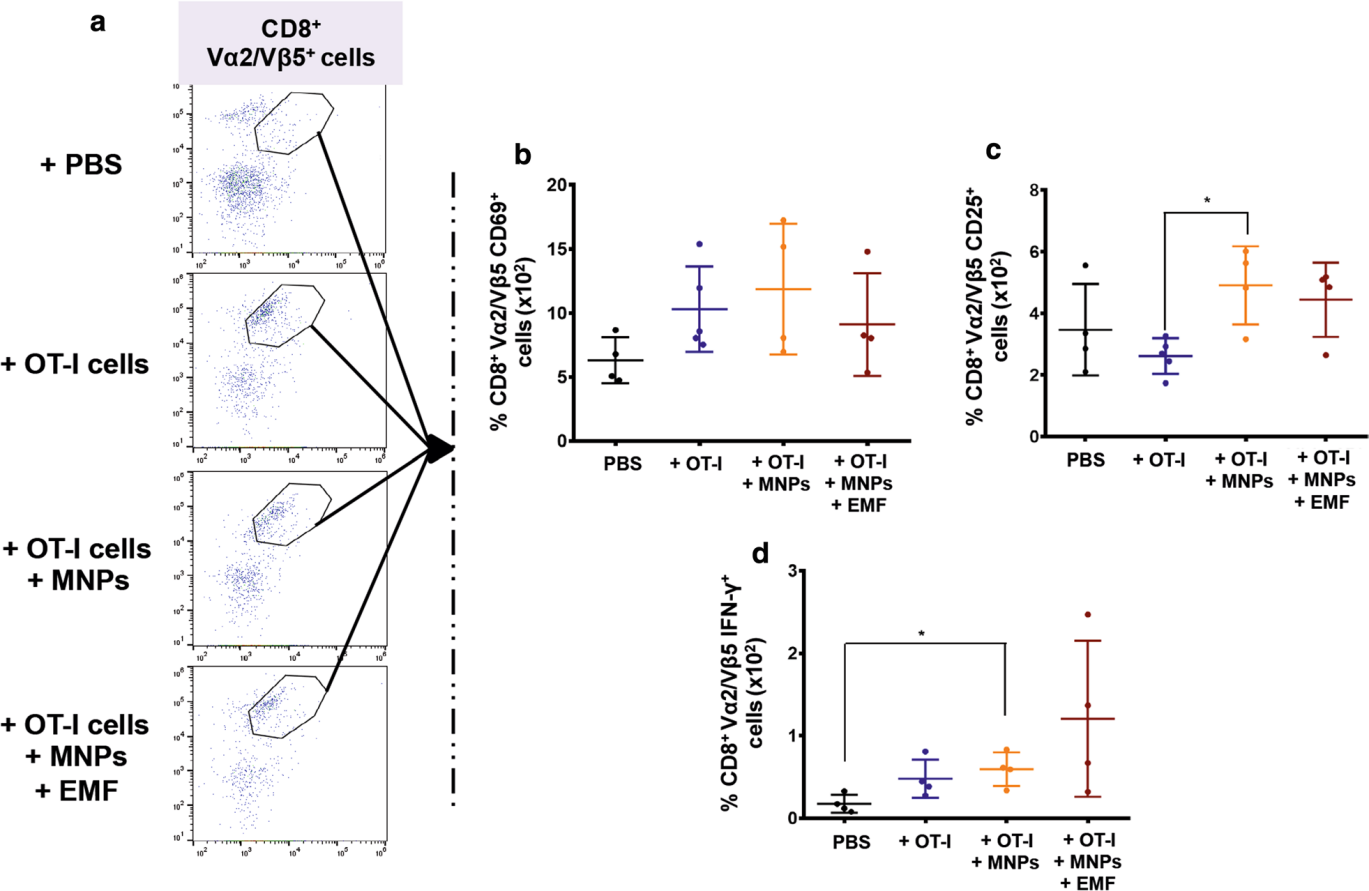
Phenotypic analysis of the CD8^+^Vα2/Vβ5^+^ T cells infiltrated in the tumour in the different groups. **a** Representative dot plots showing the percentage of Vα2/Vβ5^+^ cells within the population of live CD45^+^CD8^+^ cells. Percentage of CD8^+^ Vα2/Vβ5^+^, **b** CD69^+^, **c** CD25^+^, and **d** IFN-γ^+^ T cells. The results shown (mean ± SD) correspond to 4 or 5 mice/group, * p < 0.05, ** p < 0.01, *** p < 0.001

To see if the observed differences in tumour size and infiltration grade could be attributable to differences in the activation state of the transferred cells, we analysed the expression of different activation markers of this CD8^+^ Vα2/Vβ5^+^ T cell infiltrate and its potential response to the soluble OVA_257–264_ peptide in the tumour, analysing its ability to produce IFN-γ (Fig. 9). No significant changes were found in the percentage of CD8^+^ Vα2/Vβ5^+^ CD69^+^ T cells, although there was a slight increase in the groups in which the transfer involved OT-I CD8^+^ T cells (either in the presence or absence of APS-MNPs and EMF) in comparison to the PBS-treated group (Fig. 9b). The groups inoculated with APS-MNP-loaded OT-I CD8^+^ T cells and exposed to an EMF or not presented a higher percentage of CD8^+^ Vα2/Vβ5^+^ CD25^+^ T cells, especially in comparison to the group inoculated with OT-I CD8^+^ T cells only ((2.6 ± 0.5) × 10^−2^ % in the group transferred with OT-I CD8^+^ T cells vs (4.9 ± 1.1) × 10^−2^ % and (4.5 ± 1.0) × 10^−2^% in the groups treated with APS-MNP-loaded OT-I CD8^+^ T cells in the absence or presence of an EMF, respectively) (Fig. 9c). To perform an ex vivo analysis of the response of these tumour-infiltrating CD8^+^ Vα2/Vβ5^+^ T cells, tumours were removed from treated animals, disaggregated, and restimulated with the OVA_257–264_ peptide. Then, IFN-γ production by CD8^+^ Vα2/Vβ5^+^ T cells was measured by flow cytometry. In this case, all the groups transferred with OT-I CD8^+^ T cells (either in the presence or absence of APS-MNPs and EMF) presented a higher percentage of CD8^+^ Vα2/Vβ5^+^ IFN-γ^+^ T cells compared to the group treated with PBS; although not statistically significant due to the high variability, this increase was more evident in the group treated with APS-MNP-loaded OT-I CD8^+^ T cells ((0.5 ± 0.2) × 10^−2^ %, (0.6 ± 0.2) × 10^−2^ %, and (1.2 ± 0.8) × 10^−2^ % in the groups treated with OT-I CD8^+^ T cells, APS-MNP-loaded OT-I CD8^+^ T cells, and APS-MNP-loaded OT-I CD8^+^ T cells exposed to an EMF, respectively, vs (0.2 ± 0.1) × 10^−2^ % in the PBS group) (Fig. 9d).

### EMF application promotes accumulation in the tumour-draining LN of APS-MNP-loaded OT-I CD8^+^ T cells with an activated profile

Additionally, since T cells activated in LNs can be further stimulated and either remain as CD8^+^ memory T cells or migrate to the tumour microenvironment [13, 53] and LNs are the most common sites of solid-tumour metastases [40], we further examined the biodistribution of these OT-I CD8^+^ T cells in different secondary lymphoid organs by analysing the presence of CD8^+^Vα2/Vβ5^+^ T cells in the tumour-draining LN, the primary site of metastasis initiation, as well as a LN distal to the tumour and the spleen. For these cases, a different gating strategy was followed (see Additional file 1: Fig. S3).

**Fig. 10:**
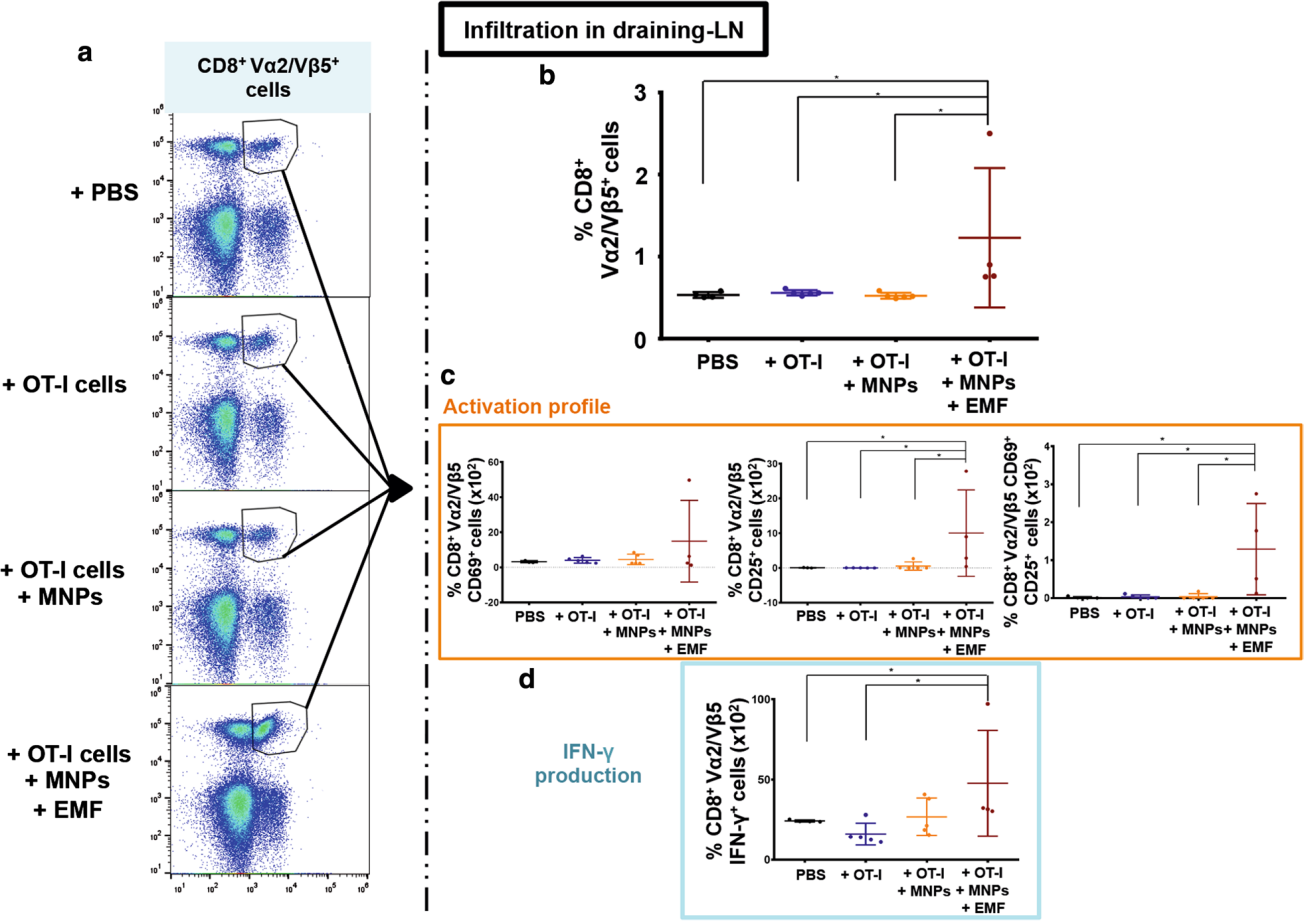
CD8^+^Vα2/Vβ5^+^ T cell infiltration in the tumour-draining LN in the different groups. **a** Representative dot plots showing the percentage of Vα2/Vβ5^+^ cells within the living CD3^+^CD8^+^ cell population. **b** Percentage of CD8^+^ Vα2/Vβ5^+^ T cells within living CD45^+^ cells within the tumour-draining LN in the different treatment groups. **c** Percentage of CD8^+^ Vα2/Vβ5^+^ CD69^+^, CD25^+^, and CD69^+^CD25^+^ T cells in the tumour-draining LN in the different treatment groups. **d** Percentage of CD8^+^ Vα2/Vβ5^+^ IFN-γ^+^ T cells in the tumour-draining LN in the different treatment groups. The results shown (mean ± SD) correspond to 4 or 5 mice/group, * p < 0.05, ** p < 0.01, *** p < 0.001

The analysis of the CD8^+^ Vα2/Vβ5^+^ T cells infiltrating the tumour-draining LN revealed that the group inoculated with APS-MNP-loaded OT-I CD8^+^ T cells and exposed to the EMF had the highest value compared to the rest of the groups (1.15 ± 0.9% in the PBS group vs 0.53 ± 0.04%, 0.56 ± 0.03%, and 0.53 ± 0.04% in the PBS, OT-I CD8^+^ T cell, and APS-MNP-loaded OT-I CD8^+^ T cell groups, respectively) (Fig. 10a, b).

The CD8^+^ Vα2/Vβ5^+^ T cells infiltrating the LN of the EMF-exposed group also showed higher expression of the activation markers CD25 ((10.04 ± 10.75) × 10^−2^% in the PBS group vs (0.07 ± 0.06) × 10^−2^%, (0.03 ± 0.02) × 10^−2^%, and (0.55 ± 1.07) × 10^−2^% in the groups of PBS, OT-I CD8^+^ T cells, and APS-MNP-loaded OT-I CD8^+^ T cells) and CD25 together with CD69 ((1.29 ± 1.04) × 10^−2^% vs (0.02 ± 0.02) × 10^−2^%, (0.03 ± 0.04) × 10^−2^%, and (0.04 ± 0.07) × 10^−2^% in the PBS, OT-I CD8^+^ T cell, and APS-MNP-loaded OT-I CD8^+^ T cell groups) (Fig. 10c). In addition, the percentage of CD8^+^ Vα2/Vβ5^+^ IFN-γ^+^ T cells generated in response to restimulation with the OVA_257–264_ peptide was higher in this group compared to the group inoculated only with PBS or OT-I CD8^+^ T cells ((47.8 ± 28.5) × 10^−2^% vs (24.4 ± 0.6) × 10^−2^% and (17.1 ± 6.1) × 10^−2^% in the PBS and OT-I CD8^+^ T cell groups, respectively) (Fig. 10d).

In similar fashion, the presence of CD8^+^ Vα2/Vβ5^+^ T cells in an LN located distal to the tumour was analysed. This analysis showed no significant differences in the percentage of CD8^+^ Vα2/Vβ5^+^ T cells infiltrating the different groups (Additional file 1: Fig. S4A, B). In addition, no significant changes were found in the expression of the activation markers CD69 and CD25 (Additional file 1: Fig. S4C) or in the production of IFN-γ after restimulation with the OVA_257–264_ peptide (Additional file 1: Fig. S4D). It was further observed that the activation profile was lower in this case compared to the profile found in the tumour-draining LN (Fig. 10c, d; Additional file 1: Fig. S4C, D).

**Fig. 11:**
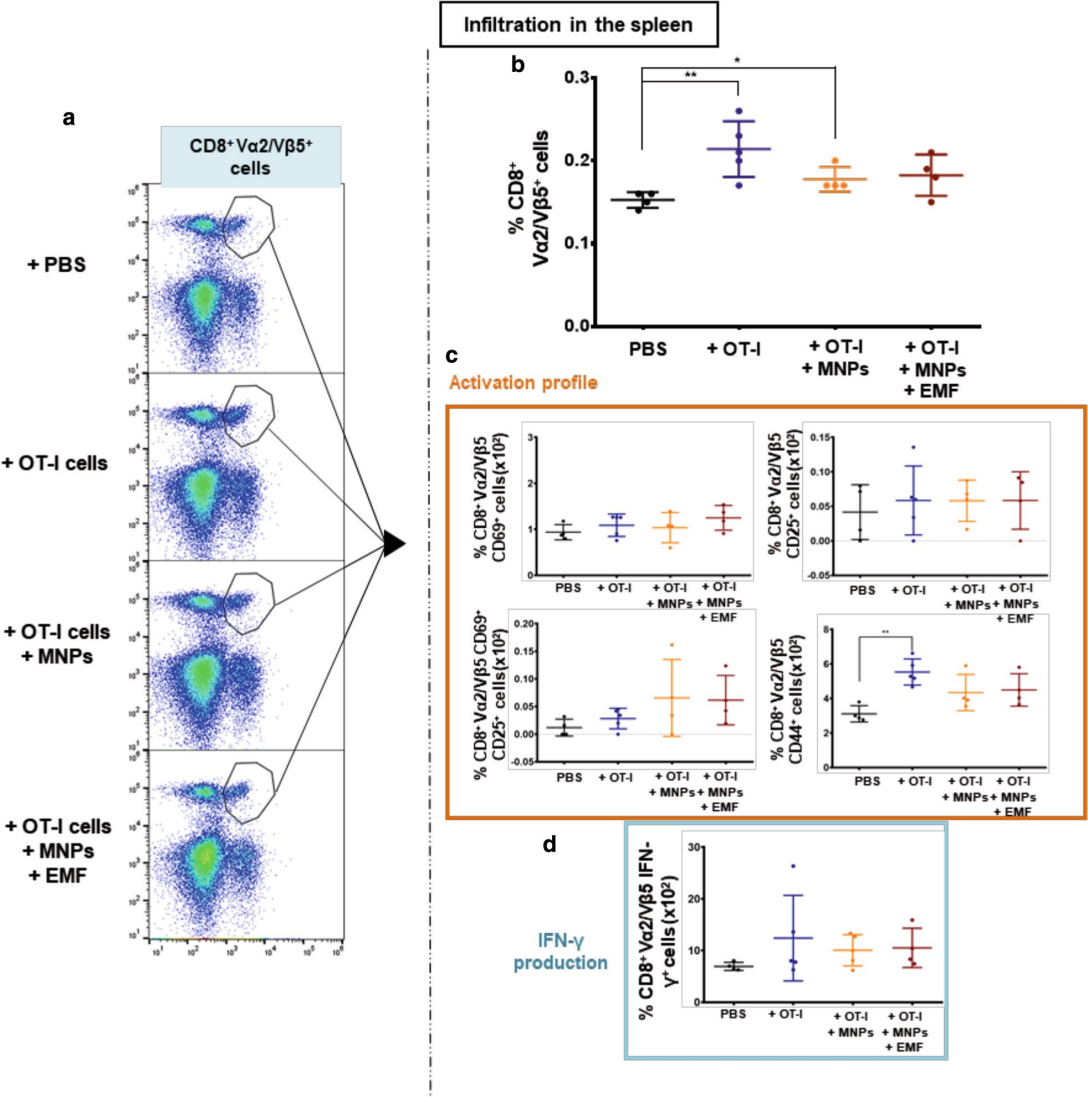
CD8^+^Vα2/Vβ5^+^ T cell infiltration in the spleen for the different groups. **a** Representative dot plots showing the percentage of Vα2/Vβ5^+^ cells within the living CD3^+^CD8^+^ cell population. **b** Percentage of CD8^+^ Vα2/Vβ5^+^ T cells within living CD45^+^ cells in the spleen for the different treatment groups. **c** Percentage of CD8^+^ Vα2/Vβ5^+^ CD69^+^, CD25^+^, CD69^+^CD25^+^, and CD44 ^+^ T cells in the spleens of animals included in the different treatment groups. **d** Percentage of CD8^+^ Vα2/Vβ5^+^ IFN-γ^+^ T cells in the different treatment groups. The results shown (mean ± SD) correspond to 4 or 5 mice/group, *p < 0.05, **p < 0.01, ***p < 0.001

The presence of CD8^+^ Vα2/Vβ5^+^ T cells in the spleen was also analysed. This analysis showed that the spleens of the mice inoculated with OT-I CD8^+^ T cells or APS-MNP-loaded OT-I CD8^+^ T cells had a higher number of CD8^+^ Vα2/Vβ5^+^ T cells (0.21 ± 0.03% and 0.18 ± 0.02%, respectively) in comparison with the group treated with PBS only (0.15 ± 0.01%) (Fig. 11a, b). In addition, no significant changes were found in the expression of the activation markers CD69 and CD25 (Fig. 11c) or in IFN-γ production after restimulation with the OVA_257–264_ peptide (Fig. 11d). Furthermore, the expression of the activation marker CD44 was higher compared to the group treated with PBS; this difference was more significant in the group treated with OT-I CD8^+^ T cells ((3.1 ± 0.5) × 10^−2^% in the PBS group vs (5.5 ± 0.7) × 10^−2^%, (4.3 ± 0.9) × 10^−2^%, and (4.5 ± 0.8) × 10^−2^% in the groups receiving PBS, OT-I CD8^+^ T cells, and APS-MNP-loaded OT-I CD8^+^ T cells) (Fig. 11c).

**Fig. 12:**
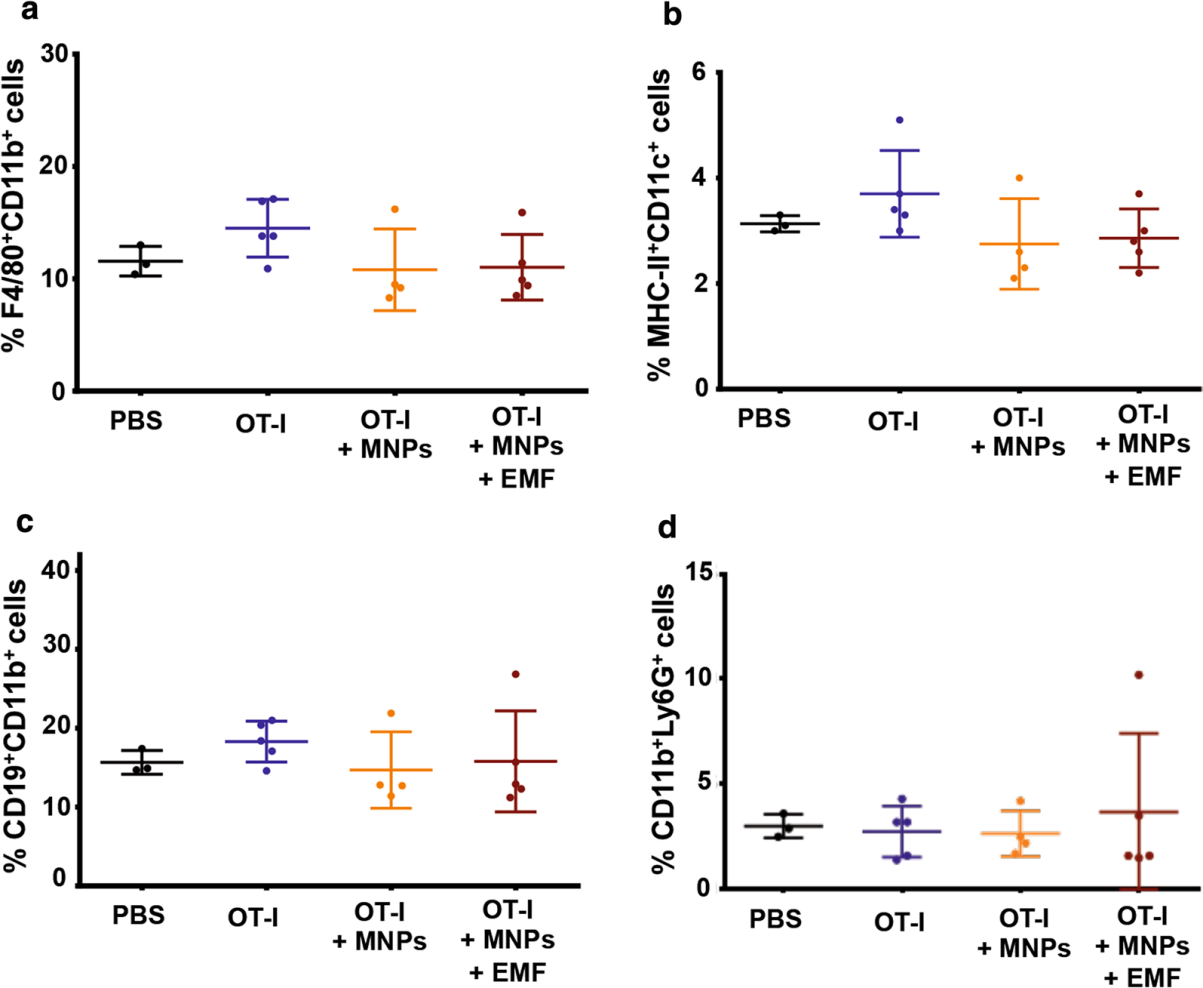
Tumour infiltration by other immune cells in the different treatment groups. Quantification of the results obtained by flow cytometry regarding infiltration by **a** macrophages, **b** dendritic cells, **c** B cells, and **d** neutrophils in the tumour region. The results shown (mean ± SD) correspond to 4 or 5 mice/group, * p < 0.05, ** p < 0.01, *** p < 0.001

Finally, we investigated the infiltration of other immune-cell types into the tumour by means of flow-cytometry analysis of the presence of macrophages (F4/80^+^CD11b^+^ cells), dendritic cells (DCs) (MHC-II^+^CD11c^+^ cells), B cells (CD19^+^CD11b^+^ cells), and neutrophils (CD11b^+^Ly6G^+^ cells). No significant differences were detected in the infiltration of macrophages, DCs, B cells, or neutrophils in the tumour tissue in the different treatment groups (Fig. 12a–d).

## Dicussion

To assess the combined use of MNPs and EMFs to magnetically retain tumour-specific CD8^+^ T cells in a solid tumour and in its draining LNs, we synthesized and characterized different MNPs, and studied their possible influence over the functionality of these CD8^+^ T cells as well as their magnetic retention both in vitro and in vivo. The MNPs used in this study presented an average core diameter of 12.5 nm and different coatings. These MNPs have been used before with primary murine T cells as well as with the human T cell line Jurkat and no significant effects were found on the basic biological aspects of these T cell models after MNP treatment. These MNP-loaded primary T cells could also be magnetically retained in LNs [38]. In the present work, we used antitumour effector CD8^+^ T cells from OT-I mice to assess the possibility of magnetically target and accumulate tumour-specific CD8^+^ T cells in solid tumours as well as in the tumour-draining LN for cancer treatment and potentially reduce metastasis spreading.

MNP treatment did not cause any toxicity in the tumour-specific CD8^+^ T cells in the different conditions tested. Furthermore, the APS-MNPs showed the greatest association with these cells. These results are in accordance with those obtained with the human T cell line Jurkat and primary murine T cells [38]. For this reason, we selected APS-MNPs for subsequent experiments. It is generally assumed that positively charged NPs interact to a greater extent with cell membranes, possibly due electrostatic interactions [54–58]. In our case, positive MNPs (APS-MNPs) interacted more with the membrane of the lymphoid cells, as described in the literature. MNP uptake or association with the cell membrane increased in T cells after in vitro stimulation, suggesting that the activation state of T cells could influence their MNP-uptake capacity [59]. In fact, previous results showed that unprimed primary T cells were associated with these MNPs to a lesser extent when compared to the primed CD8^+^ T cells [38].

Microscopy studies showed that APS-MNPs were attached to the surface of OT-I CD8^+^ T cells. These results are also in accordance with what we observed in a previous study, where Jurkat or primary murine T cells were incubated with APS-MNPs, and APS-MNPs remained on the cell surface in close contact with the cell membrane [38].

The analysis of the cell-surface phenotype after MNP treatment showed that the association of OT-I CD8^+^ T cells with APS-MNPs did not produce significant differences in the expression levels of important surface markers. These data are consistent with previous studies showing that MNPs may not significantly affect the expression levels of T cell surface markers [60]. Nevertheless, other studies have shown that MNPs can induce some intracellular activation markers in certain cases [61, 62], although this depends mainly on NP design [63].

The main effector functions of such CD8^+^ T cells as OT-I CD8^+^ T cells are the induction of cytotoxicity in infected or transformed cells as well as the production of a range of cytokines like IFN-γ. Very little is known about the effect of MNPs on the cytotoxic functions of T cells. What is known comes from studies designed to evaluate the use of MNPs to track cytotoxic T cells by magnetic resonance imaging, analysing some functional aspect of these cells after their association with the MNPs. These studies suggested that proliferation in response to specific antigens, as well as the cytotoxic capacity against certain cell targets or the production of certain cytokines, both in human and murine T cells, do not seem to be affected by MNPs in vitro [60, 64, 65]. Nonetheless, certain defects in the cytolytic capability and cytokine production of these cells has also been described, but only at very high doses of MNPs (> 500 μg Fe/ml) [59]. Although the reports mentioned above suggest that T cell effector functions may not be affected by interaction with MNPs, we decided to systematically analyse the effector functions of OT-I CD8^+^ T cells after their association with APS-MNPs.

The results in this work indicate that the presence of APS-MNPs in the cell membranes of cytotoxic CD8^+^ T cells did not affect their ability to physically interact with and adhere to their cell targets.

We also verified that the presence of APS-MNPs did not affect the lytic capacity of OT-I CD8^+^ T cells. OT-I CD8^+^ T cells can be activated and are able to lyse target cells in vitro, even in association with APS-MNPs, as previously described [36, 59, 60, 66–68].

Another essential aspect concerning the functionality of cytolytic cells is the ability to produce pro-inflammatory cytokines such as IFN-γ [48]. In the present work, OT-I CD8^+^ T cells were able to produce similar levels of IFN-γ after activation by non-specific or specific stimuli in the presence or absence of APS-MNPs. These results are in line with previous studies [59, 64, 65]. Furthermore, OT-I CD8^+^ T cells were able to adhere and transmigrate in vitro across an endothelium after MNP treatment. Therefore, the results obtained here support those of several studies showing that effector T cells associated with MNPs could migrate, infiltrating the tumour in vivo without difficulty [43, 60, 66–70].

The evaluation of the in vitro magnetic retention of APS-MNP-loaded OT-I CD8^+^ T cells in a dynamic flow system using magnets showed that the displacement of APS-MNP-loaded OT-I CD8^+^ T cells toward the EMF was similar to the one observed in a previous study using primary T cells [38], indicating that this magnetic retention increases at higher quantities of MNPs associated with the cells and at higher degrees of magnetic field magnitude. Other studies also reported that these two parameters are critical for this magnetic targeting to occur [35, 71].

The present study lends further support to the notion that an EMF is capable of promoting in vitro retention of lymphoid cells associated with APS-MNPs in the presence of flow forces resembling those present in blood capillaries.

Finally, our data indicate that APS-MNPs did not crucially affect the chemotactic response of OT-I CD8^+^ T cells and that adequate application of an EMF could facilitate the migration of APS-MNP-loaded OT-I CD8^+^ T cells in some way.

As seen throughout this first set of in vitro tests, the association between OT-I CD8^+^ T cells and APS-MNPs did not crucially affect the functionality of these cells. Therefore, the combination of APS-MNPs and EMFs was evaluated to determine whether this system could be used to promote the specific accumulation of these cytolytic cells in an in vivo model and thus optimise cell-transfer protocols. So far, a few studies have used this strategy, though they have accumulated stem and mesenchymal cells, macrophages, or dendritic cells and for the purpose of tissue regeneration and to treat autoimmune disorders [31–35]. Only one study has investigated magnetic targeting of lymphoid cells, specifically, a natural killer cell line [36].

In order to evaluate the efficacy of combined use of MNPs and EMFs in the transfer of cells with antitumor activity for cancer treatment, we chose a murine syngeneic tumour model, where the implanted EG7-OVA tumour cells express an OVA antigen that can be specifically recognised by OT-I CD8^+^ T cells. It is known that higher infiltration of OT-I CD8^+^ T cells correlates with lower tumour growth [42–44]. For this reason, using this model allowed us to test whether the application of an EMF in close contact with the tumour could increase tumour infiltration and accumulation of OT-I CD8^+^ T cells due to magnetic retention. Subsequently, we also analysed the capacity of these OT-I CD8^+^ T cells to remain in the tumour-draining LN, a potential site for solid-tumour metastases [40]. The group inoculated with APS-MNP-loaded OT-I CD8^+^ T cells that had been exposed to an EMF during and after the cell transfer showed intermediate growth while the groups treated with OT-I CD8^+^ T cells without APS-MNPs or with APS-MNP-loaded OT-I CD8^+^ cells showed the lowest tumour growth. Therefore, it seems that even though APS-MNP-loaded OT-I CD8^+^ T cells maintain their in vivo antitumour capacity, the application of an EMF for magnetic targeting has an apparently negative effect on the capacity of the T cells to control tumour growth.

To understand why application of an EMF appeared to reduce the capacity of APS-MNP-loaded OT-I CD8^+^ T cells to control tumour growth, we performed a detailed analysis of the different cell populations found in the tumour as well as different secondary lymphoid organs. The group treated with APS-MNP-loaded OT-I CD8^+^ T cells and exposed to EMF presented an intermediate value compared to the rest of the treatment groups. If we take into account the differences in tumour size observed, there is a clear correlation between smaller tumour size and greater proportion of infiltrating OT-I CD8^+^ T cells, as previously described for this model [42–44].

The results obtained here showed that the presence of APS-MNPs on the OT-I CD8^+^ T cell surface did not affect their in vivo migration and infiltration into the tumour or their antitumor properties, since the result was very similar to that achieved by the OT-I CD8^+^ T cells in the absence of MNPs. These results are consistent with those of previous studies showing that MNPs do not interfere with the ability of effector T cells to migrate and infiltrate the tumour and subsequently eliminate tumour cells [43, 68]. However, when an EMF was applied to the tumour area to study its potential to favour the retention and infiltration of the APS-MNP-loaded OT-I CD8^+^ T cells by increasing the adhesion time in the region, no improvement was achieved. In fact, this group showed intermediate tumour-growth control, that is, its efficacy was between that obtained after treatment with PBS only, as a negative control, and the groups treated with OT-I CD8^+^ T cells or with APS-MNP-loaded OT-I CD8^+^ T cells.

The analysis of the infiltration of CD8^+^ Vα2/Vβ5^+^ T cells correlated with the clinical result obtained, since the groups treated exclusively with OT-I CD8^+^ T cells (with and without APS-MNPs) evidenced higher infiltration, while the group that was additionally exposed to an EMF had intermediate infiltration, which is to say, a rate that was between these two groups and the one treated with PBS only. Additionally, the group treated with PBS presented certain basal levels of CD8^+^ Vα2/Vβ5^+^ T cells, possibly due to the random reorganisation of the TCRs and the generation of clones that can recognise these tumour cells. An analysis of the activation of these cells in the different groups showed that, in general, the cells that had infiltrated the tumours of all the groups treated with OT-I CD8^+^ T cells seemed to present a higher activation state (i.e., higher percentage of CD69^+^ and CD25^+^ cells) and to produce more IFN-γ after restimulation with the OVA_257–264_ peptide.

T lymphocytes migrate to LNs to become activated by antigen-presenting cells so that they can migrate and lyse the tumour effectively. In addition, T cells activated in LNs can be further stimulated to divide, produce effector cytokines, and either remain as CD8^+^ memory T cells or migrate to the tumour microenvironment [13, 53]. Furthermore, LNs are the most common sites of solid-tumour metastases, and tumours usually produce alterations in order to establish metastatic lesions and prevent functional T cells from reaching the LN and mounting an antitumoral immune response [40]. For these reasons, we examined the biodistribution of these OT-I CD8^+^ T cells in different secondary lymphoid organs by analysing the presence of CD8^+^Vα2/Vβ5^+^ T cells in the tumour-draining LN, the primary site of metastasis initiation, as well as a LN distal to the tumour and the spleen.

The analysis of the infiltration into the tumour-draining LN showed that the group treated with APS-MNP-loaded OT-I CD8^+^ T cells and exposed to EMF presented greater infiltration in the tumour-draining LNs of CD8^+^ Vα2/Vβ5^+^ T cells with an activated profile. This indicates that a fraction of the transferred APS-MNP-loaded OT-I CD8^+^ T cells infiltrated the tumour-draining LN and remained there 14 days after cell transfer. This could be due to the fact that, since the LN and the tumour are very close, exposure to an EMF promoted the retention of transferred cells in the LN, a location offering easier access for these cells. This magnetic retention of activated APS-MNP-loaded effector CD8^+^ T cells in the tumour-draining LN could hold potential to stop cancer from spreading through the LNs, although this hypothesis needs to be tested. Besides, in previous studies we observed that APS-MNP-loaded naïve T cells infiltrated LNs more and tended to remain there [38].

The absence of differences in the infiltration of CD8^+^ Vα2/Vβ5^+^ T cells in a LN located distal to the tumour as well as their low activation profile indicates that the accumulation of APS-MNP-loaded OT-I CD8^+^ T cells in the tumour-draining LN seen in the EMF-exposed group was specific and mainly due to the EMF.

There are several possible explanations as to why APS-MNP-loaded OT-I CD8^+^ T are retained in the LN instead of exiting the LN and infiltrating the tumour. As previously discussed, the reduced velocity of the T cells due to the MNPs and the EMF could prolong their interaction with the vasculature of the lymphoid tissue and facilitate retention of these cells. In addition, the tendency with which cells carrying MNPs to aggregate [72] could impede egress and contribute to their inability to abandon the LN. Along these lines, the different microscopy studies performed during the present work revealed some cell aggregation in vitro after 2 h of MNP treatment (Additional file 1: Fig. S5), which was not as frequent in the absence of MNP. However, when tumours in mice were removed 14 days after the different treatments and CD8^+^ T cells were analysed by immunohistochemistry, we did not observe that the presence of APS-MNP or the application of an EMF induced more aggregation in tumor-infiltrating CD8^+^ T cells (Additional file 1: Fig. S6). Similarly, when APS-MNP-loaded T cells were transferred to mice and EMF-exposed to promote their retention in a certain lymph node, no special distribution was observed (Fig. 8a [38]). This shared accumulation of activated tumour-specific CD8^+^ T cells both in the tumour and in the proximal LN is less efficient in terms of reducing tumour size compared to APS-MNP transferred without an EMF, but the capacity of this approach to induce long-term retention of these cells in the tumour-draining LN could protect against metastasis spreading via the LNs, although this theory needs to be further analysed. Moreover, this approach could be combined with other strategies to attack the tumour from different points and obtain an optimal antitumor immune response.

## Conclusions

In this work we explore whether the combined use of APS-MNPs, which can be directly attached to the cell membrane of T cells, and the application of EMFs near a tumour in a mouse model of cancer in which the tumour expresses an antigen recognised by the transferred T cells could promote the accumulation of tumour-specific CD8^+^ T cells in the tumour region in ACT therapies.

APS-MNP-loaded OT-I CD8^+^ T cells preserved both their in vitro and in vivo functionality, and application of an EMF promoted cell retention under flow conditions in vitro. APS-MNPs attached to the cell membrane of adoptively transferred OT-I CD8^+^ T cells together with the application of an EMF in close proximity to the tumour during cell transfer reduced the infiltration of these transferred cells into the tumoral region but simultaneously promoted the retention of activated tumour-specific OT-I CD8^+^ T cells in the tumour-draining lymph nodes. Furthermore, the percentage of activated tumour-specific OT-I CD8^+^ T cells infiltrating the tumour-draining lymph nodes 14 days after cell transfer was higher with the application of an EMF in close proximity to the tumour. Previous results from our group showed that APS-MNPs promoted retention, which could cause the transferred T cells to become scattered between the tumour and the LN.

Nanotechnology has already brought great improvement to certain therapies. Controlling immune-cell traffic would likely bring substantial improvement to the treatment of various diseases, although this area is still in its infancy due to the complex regulatory processes at work on different levels.

## Methods

### MNP synthesis and characterisation

The synthesis and characterisation of the different MNPs used in this study have been described previously [38]. Briefly, iron oxide cores were prepared by following the Massart coprecipitation protocol [73]; these cores were then coated with DMSA, APS, or dextran (6 kDa) in accordance with previously described procedures [74]. After this, physico-chemical characterisation of the coated MNPs was carried out by various analytical procedures. Hydrodynamic diameter and Z-potential were measured by DLS, and presence of the coating as well as its percentage were studied by infrared spectroscopy and thermogravimetric analyses, respectively. MNP morphology analyses were performed using transmission electronic microscopy and the magnetic properties of MNPs were measured in a vibrating sample magnetometer.

### Mice

C57BL/6-Tg(TcraTcrb)1100Mjb mice (OT-I) were kindly provided by Dr. I. Mérida (CNB), and these were used to start our own colony. These transgenic mice express the transgenic OT-I TCR (Vα2 and Vβ5), which recognises OVA_257–264_ (SIINFEKL), an ovalbumin (OVA) peptide specific for the MHC-I molecule H-2 Kb [41, 75]. These mice were phenotyped by flow cytometry using an antibody that recognises Vα2, which is specific for this type of receptor. Female C57BL/6 mice that were 5 to 7 weeks old were purchased from Harlan Laboratoriesand used to generate a syngeneic tumour model. Both mice strains were maintained in the CNB animal facility and handled according to the recommendations of the CNB-CSIC institutional ethics committee. The procedures involving animals were approved by the ethics committee for animal experimentation of the CSIC and by the Division of Animal Protection of the Comunidad de Madrid in compliance with national and European Union legislation.

### Cell culture

The murine endothelial cell line SVEC4-10 (ATCC: CRL-2181) was cultured in DMEM with 10% FBS, 2 mM l-glutamine, 1 mM sodium pyruvate, and 100 U/ml penicillin/streptomycin (P/S) under standard culture conditions (37 °C, 5% CO_2_, 90% relative humidity). The tumour-cell line EG7-OVA (ATCC: CRL2113) was cultured in RPMI1640 with 10% FBS, 2 mM l-glutamine, 1 mM sodium pyruvate, 50 μM 2-mercaptoethanol, 10 mM HEPES, non-essential amino acids 1X, 100 U/ml P/S (complete RPMI medium), and in the presence of 0.4 mg/ml geneticin (G418, ThermoFisher Scientific) to select only plasmid-carrying cells, all under standard culture conditions.

OT-I CD8^+^ T cells were purified from spleen and LN-cell suspensions obtained from OT-I transgenic mice (Jackson Laboratories). These cell suspensions (5 × 10^6^ cells/ml) were cultured for 2 days in complete RPMI medium supplemented with the soluble OVA_257–264_ peptide (synthesised by the CNB Proteomics Service) at 10 pM in order to activate the OT-I CD8^+^ T cells present. After 2 days, most of the inactive cells died, and only those that had been activated survived. At this point the culture medium was replaced by complete RPMI medium supplemented with murine recombinant IL-2 (50 U/ml, Peprotech) at a cell density of 0.2–0.4 × 10^6^ cells/ml, and activated OT-I CD8^+^ T cells were allowed to expand for 3 days. The percentage of OT-I CD8^+^ T cells (CD3^+^CD8^+^) as well as their activation profile, as measured by the expression of the early activation marker CD69 and expression of the late activation marker CD25, was checked by flow cytometry at day 0 and day 5–6 after activation, obtaining around 90% to 95% of OT-I CD8^+^ T cells after expansion. At this point they were used in the corresponding experiments.

### Cell viability, MNP uptake, and staining assays

Cell viability was examined by two methods. In the Alamar Blue assay (Invitrogen), OT-I CD8^+^ T cells were cultured with different MNP concentrations for 24 h, after which Alamar Blue was added and then incubated for 4 h, at which point fluorescence was measured. For FITC-annexin V/propidium iodide staining, cells were processed using the Annexin V-PI apoptosis assay kit following the manufacturer’s protocol (Life Technologies) and analysed by flow cytometry.

To analyse MNP uptake by ICP-OES (inductively coupled plasma—optical emission spectrometry*)*, OT-I CD8^+^ T cells (10^7^ cells/ml) treated with MNPs (150 µg Fe/ml) for 2 h were sequentially digested at 90 °C with HNO_3_ 63% and then with H_2_O_2_.

For iron staining, OT-I CD8^+^ T cells were fixed in 4% paraformaldehyde (PFA) for 15 min after being treated with MNPs, permeabilised, stained with Prussian blue staining for 20–30 min and counterstained with a 0.5% solution of neutral red for 1 min. The samples were then mounted to finally acquire images on an Olympus IX70 inverted bright field microscope with 63× or 100× oil-immersion objectives. For dark-field confocal microscopy, LysoTracker Red DND-99 (Life Technologies), Alexa Fluor 647-wheat-germ agglutinin (Life Technologies), and DAPI were used to stain the OT-I CD8^+^ T cells after MNP treatment. Finally, samples were mounted in Fluoromount-G (Southern Biotec) and images were acquired using a confocal multispectral Leica TCS SP5 system with a 63 ×/1.4 NA oil-immersion objective. For TEM microscopy, OT-I CD8^+^ T cells were fixed at room temperature and processed by the Transmission Electron Microscopy Service at the National Centre for Biotechnology (CNB-CSIC, Madrid, Spain). Images were acquired with a JEOL JEM 1011 transmission electron microscope at different magnifications.

### Flow cytometry

Where necessary, the “LIVE/DEAD Fixable Dead Cell Stain Kit (488 nm excitation)” (ThermoFisher Scientific) was used following the manufacturer’s instructions.

The following primary anti-mouse antibodies were used: anti-CD90.2 (53–2.1), -CD3 (17A2), -CD8 (53–6.7), -CD27 (LG.7F9), -CD127 (A7R34), -F4/80 (BM8), -CD19 (eBio D3), -Vα2 (B20.1), -CD69 (H12F3), -IFN-γ (XMG1.2), and -CD62L (Mel-14) from eBioscience; anti-CD11c (H13), -Ly6G (148), -Vβ5 (MR9 -4), -CD25 (7D4), -Vα2 (B20.1), -CD69 (H12F3), -CD25 (PC61), and -CD62L (Mel-14) from Pharmingen; and anti-CD8 (53-2.1), -CD44 (KM201, Beckman), -CD45 (30-F11), -IA/IE (M5/114.15.2), -CD11b (M1/70), -NKp46 (29A.1), -CD44 (IM7), and -CD4 (GK1.5) from Biolegend.

When necessary, after labelling with a biotinylated primary antibody, secondary staining with fluorophore-conjugated streptavidin followed. Data were acquired on a FC500 flow cytometer or in a Gallios flow cytometer and analysed with FlowJo or Kaluza software.

To perform intracellular protein labelling, cells were permeabilised with a 0.5% saponin solution in PBS staining. First, they were washed with 150 μl/well of this solution and subsequently labelled with the corresponding antibodies diluted for 30 min at 4 °C in this solution at the appropriate concentration. Finally, these cells were washed and resuspended in PBS for analysis by flow cytometry. (Note: in this case, cells were also incubated in the presence of brefeldin A or monensin to inhibit protein transport and to detect intracellular proteins.)

### Conjugation assays

In vitro expanded OT-I CD8^+^ T cells treated with APS-MNPs or not, as well as the corresponding cellular target (EG7-OVA cells), were stained with the fluorescent labelling kits of cells in red (PKH26) or green (PKH67) (both from Sigma-Aldrich), alternating labelling between the effector and target cells in the different experiments. OT-I CD8^+^ T cells and their corresponding target cells were co-incubated for varying periods of time at a 1:1 ratio under standard incubation conditions, after which they were fixed in 1% PFA and analysed by flow cytometry. Each condition was performed in duplicate. The positive events in both colours were considered to be conjugated between the OT-I CD8^+^ T cell and its target and the percentage of conjugated OT-I CD8^+^ T cells was calculated as follows: (% conjugated OT-I CD8^+^ T cells/ % total OT-I CD8^+^ T cells) × 100%.

### Degranulation assays

We inclubated 2 × 10^5^ in vitro expanded OT-I CD8^+^ T cells that (treated with APS-MNPs) with EG7-OVA cells at a 1:2 ratio (OT-I CD8^+^ T cells: EG7-OVA cells) in the presence of 10 μg/ml of monensin (Sigma-Aldrich) and 5 μl of the CD107a antibody conjugated with FITC (or its corresponding control). After 4–5 h of incubation under standard conditions, CD3 and CD8 markers were labelled for cytometry. The percentage of CD3^+^CD8^+^CD107a^+^ cells was analysed by flow cytometry.

### Cytotoxicity assays

In vitro expanded OT-I CD8^+^ T cells treated with APS-MNPs or not were co-incubated with their corresponding targets (EG7-OVA cells), which had been previously stained with the green fluorescent label kit (PKH67) (Sigma-Aldrich) at different ratios in a total volume of 200 μl for 5 h under standard incubation conditions in 96-well U-bottom plates. Each condition was performed in duplicate. The reaction was stopped by adding 250 μl of cold PBS staining and the plates were placed in ice. Fifteen μl of PI was added to each well just before being analysed by flow cytometry. The target cells were found to be positive in the FL1 channel. The percentage of specific lysis was calculated for each target cell as follows: % Lysis = (% dead target cells − % spontaneous death)/(100 −  % spontaneous death) × 100%. The percentage of spontaneous death was carried out in the absence of effector cells.

### Intracellular staining of IFN-γ

In vitro expanded OT-I CD8^+^ T cells treated with APS-MNPs or not were exposed to different stimuli to determine their IFN-γ production capacity by analysing intracellular IFN-γ levels using flow cytometry. To do this, 2 × 10^5^ effector cells were incubated with certain stimuli or specific targets in the presence of brefeldin A (1X, Biolegend). After 4–5 h of incubation under standard conditions, they were labelled for CD3 and CD8 and then intracellular staining was carried out. The percentage of CD3^+^CD8^+^IFN-γ^+^ cells was analysed by flow cytometry.

The different stimuli used in these assays were the following: co-incubation with EG7-OVA cells at a 1:2 ratio (OT-I CD8^+^ T cells:EG7-OVA cells) and soluble OVA_257–264_ peptide (10 µM) or 25 ng/ml PMA and 1 µg/ml ionomycin, both from Sigma-Aldrich.

### Adhesion and transmigration capacity assessment

We seeded 3–4 × 10^4^ SVEC4-10 murine endothelial cells on coverslips with a 12-mm diameter, placing these in a 24-well culture plate and cultured under standard conditions until the monolayer was formed (2–3 days). Once formed, the monolayer was activated with murine TNFα (250 U/ml, Peprotech) for 6–7 h. In addition, expanded OT-I CD8^+^ T cells were labelled with the CellTrace CFSE probe (2.5 μM, ThermoFisher Scientific) following the manufacturer’s instructions. Once marked, they were treated with different concentrations of APS-MNPs for 2 h under standard conditions, after which 5 × 10^4^ cells were seeded on the activated endothelium monolayer and incubated for 1 h (Fig. 5). Afterwards, the cells were washed with PBS, fixed with 4% PFA for 20 min, and the actin filaments were stained with phalloidin-TRITC (1: 500, Sigma-Aldrich) for 45 min at RT in the dark, in order to differentiate the monolayer in microscopic images. Finally, we washed the cells with PBS and mounted them on slides with Fluoromount-G for observation by confocal microscopy. The images were acquired with the Leica TCS SP5 confocal microscope, using the 10× and 20× lenses, sweeping the entire monolayer in 1-μm slices. ImageJ software was used to analyse the images obtained.

### Flow-chamber assays

In vitro magnetic retention assays were carried out under flow conditions in a modified channel slide (µ-Slide I Luer, 0.4-mm height, ibidi) using a two-magnet system as previously described [38]; the neodymium iron boron (NdFeB) permanent magnets (Supermagnete) used in this study are shown in Table 2. Briefly, OT-I CD8^+^ T cells (10^7^ cells/ml) were treated with MNPs (150 µg Fe/ml) or not for 2 h in standard conditions, and then washed and stained. An Olympus Inverted Microscope (model IX71) coupled to an Imaging Station cell^R under standard culture conditions was used in this assay. Shear stress was set at 0.5 dyne/cm^2^ and events were recorded every 1 s. After 60 s, the two-magnet system was applied for the next 2 min. Imaris software (Bitplane) was used to analyse displacement along the Y-axes (in the direction of the magnetic field).

### Transwell migration assay

MNP-treated and untreated OT-I CD8^+^ T cells were differentially labelled with PKH26 Red or PKH67 Green fluorescent cell-linker kits (Sigma-Aldrich) and mixed at a 1:1 ratio, after which 5 × 10^5^ cells were seeded in 0.1 ml of the appropriate medium in a transwell insert (Corning, 5-µm pore). The chemotactic gradient was created by adding the recombinant murine CXCL12 (100 nM, Peprotech) to the lower chamber. Cells migrated for 4 h and after this, cells from lower chambers were counted by flow cytometry. Cell migration was quantified and normalised for loading into an input well. In some cases, an 8 × 6-mm NdFeB permanent magnet (magnet B, Table 2) was placed below the well.

### Syngeneic tumour model

To generate a subcutaneous tumour, 0.5 × 10^6^ EG7-OVA cells in a volume of 100 μl PBS were injected into the right flank of a group of mice. Tumour growth was monitored two or three times a week, measuring tumour width (X) and length (Y) with a calibre, and calculating the tumour volume based on the following formula: (X^2^ × Y)/2. When the tumour reached 100–200 mm^3^, the treatment was started.

### Adoptive cell transfer therapy

Once 100–200-mm^3^ tumours were reached, four groups were prepared, and the different treatments were administered: 100 µl PBS (treatment 1), 8 × 10^6^ OT-I CD8^+^ T cells (treatment 2), 8 × 10^6^ MNP-loaded OT-I CD8^+^ T cells (treatment 3), and 8 × 10^6^ MNP-loaded OT-I CD8^+^ T cells with 90 min of tumour-localised EMF (treatment 4). To fix the magnet over the tumour in treatment group 4, the mice had to be previously anaesthetised by intraperitoneal injection of a mixture of ketamine (121 μg/g, Merial) and xylazine (14 μg/g, Calier). The state of the animals was monitored until they recovered completely.

The OT-I CD8^+^ T cells used were obtained from about 7 OT-I female mice; these cells had been isolated 5 days before and activated and expanded as previously indicated. Once expanded, a pool of cells was prepared, from which the different treatments for groups 2, 3, and 4 were prepared. In treatment group 4, the magnet used was the one having the highest magnetic force which gave the best results in the in vitro experiments, that is, a neodymium magnet 8 mm thick and 6 mm long (magnet B, Table 2).

In treatment group 4, in which a magnet was applied over the tumour, it was necessary to anaesthetise the animals by intraperitoneal injection of a mixture of ketamine and xylazine in order to place the magnet near the tumour. As the ethics committee authorized us to anesthetize the animals for 90 min, the time of exposure to the magnet was dictated by this time limit. We considered this time of exposure to the magnet to be sufficient to promote retention of MNP-loaded transferred cells after intravenous injection since the one-pass circulation time of blood in a mouse has been previously determined to be around 15 s [76].

Once the treatment was administered, the tumour size, as well as the state and weight of the mice, were monitored every 2–3 days for 2 weeks. After day 14, the mice were sacrified and tumours, spleens, tumour-draining LNs, and LNs distal to the tumour were collected for further analysis.

To process the tumours, a 0.3–0.4 g section was cut into small cubes of about 1 mm^3^. Then, each section was introduced into a 15-ml Falcon tube, and 2 ml of the following digestion medium was added: RPMI1640 supplemented with HEPES (20 mM, Biowest), collagenase I (2 mg/ml, Worthington Biochemical Corporation), dispase II (2.5 mg/ml, Roche), and DNase I (0.1 mg/ml, Roche). The tumour sections were incubated under stirring conditions at 37 °C for 20 min, after which they was disintegrated in a 70-μm pore filter (Corning) and finally erythrocyte lysis was carried out (5 min, RT). Subsequently, the section was washed with PBS supplemented with 5% FBS, and 5 × 10^6^ cells/sample were separated for flow cytometry staining and analysis.

Cell suspensions from spleens and LNs were obtained using a 40-μm pore filter, after which erythrocytes were lysed (5 min, RT) in the spleen samples. Subsequently, several washes were made using PBS supplemented with 5% FBS. A quantity of 2 × 10^6^ cells was separated in the spleen samples and 1 × 10^6^ cells in the LN samples was separated for flow cytometry staining and analysis.

To analyse IFN-γ production, cells were stimulated in the presence of the soluble OVA_257–264_ peptide (10 μM) and brefeldin A (1X) for 5–6 h under standard incubation conditions.

In all cases, the samples were fixed after staining and stored at 4 °C until analysis. All samples were analysed using the Gallios flow cytometer (Beckman Coulter) and Kaluza analysis software.

### Statistical analyses

The graphs and statistical analyses were made with Prism 5.0 (GraphPad) software. Two types of statistical analysis were applied according to the type of experiment: two-tailed unpaired Student *t* test or two-way ANOVA. *p < 0.05, **p < 0.01, ***p < 0.001, ****p < 0.0001.

## Additional file

**Additional file 1: Fig. S1.** Phenotyping of blood obtained from the transgenic mouse strain OT-I. **Fig. S2** OT-I CD8^+^ T cell purification and expansion. **Fig. S3** Analysis of CD8^+^Vα2/Vβ5^+^ T cell infiltration in the tumour-draining and distal LNs and in spleens. **Fig. S4** CD8^+^Vα2/Vβ5^+^ T cell infiltration of a distal LN in the different treatment groups. **Fig. S5.** Cell aggregation after MNP treatment. **Fig. S6.** Immunohistochemical analysis of CD8^+^ T cell infiltration in tumours.

## Data Availability

All data generated or analysed during this study are included in this published articles and its Additional files.
