# Supplementary material for: Magnetic targeting of adoptively transferred tumour-specific nanoparticle-loaded CD8+ T cells does not improve their tumour infiltration in a mouse model of cancer but promotes the retention of these cells in tumour-draining lymph nodes
Source: J Nanobiotechnology. 2019 Aug 6;17:87. doi: 10.1186/s12951-019-0520-0 (PMC6683429; doi:10.1186/s12951-019-0520-0)
Supplement: Supplementary file 1 — Additional file 1: Fig. S1. Phenotyping of blood obtained from the transgenic mouse strain OT-I. Fig. S2 OT-I CD8+ T cell purification and expansion. Fig. S3 Analysis of CD8+Vα2/Vβ5+ T cell infiltration in the tumour-draining and distal LNs and in spleens. Fig. S4 CD8+Vα2/Vβ5+ T cell infiltration of a distal LN in the different treatment groups. Fig. S5. Cell aggregation after MNP treatment. Fig. S6. Immunohistochemical analysis of CD8+ T cell infiltration in tumours. [file 12951_2019_520_MOESM1_ESM.docx]

**ADDITIONAL FILE 1**

**Magnetic targeting of adoptively transferred tumour-specific nanoparticle-loaded CD8^+^ T cells does not improve their tumour infiltration in a mouse model of cancer but promotes the retention of these cells in tumour-draining lymph nodes**

**Laura Sanz-Ortega^a^, Yadileiny Portilla^a^, Sonia Pérez-Yagüe^a^, Domingo F. Barber^a,^[[1]](#footnote-1)^*^**

^a^Department of Immunology and Oncology, and NanoBiomedicine Initiative, Centro Nacional de Biotecnología (CNB)-CSIC, Darwin 3, Cantoblanco, 28049 Madrid, Spain

*Address correspondence to [dfbarber@cnb.csic.es](mailto:dfbarber@cnb.csic.es)

**SUPPLEMENTARY FIGURES**

**
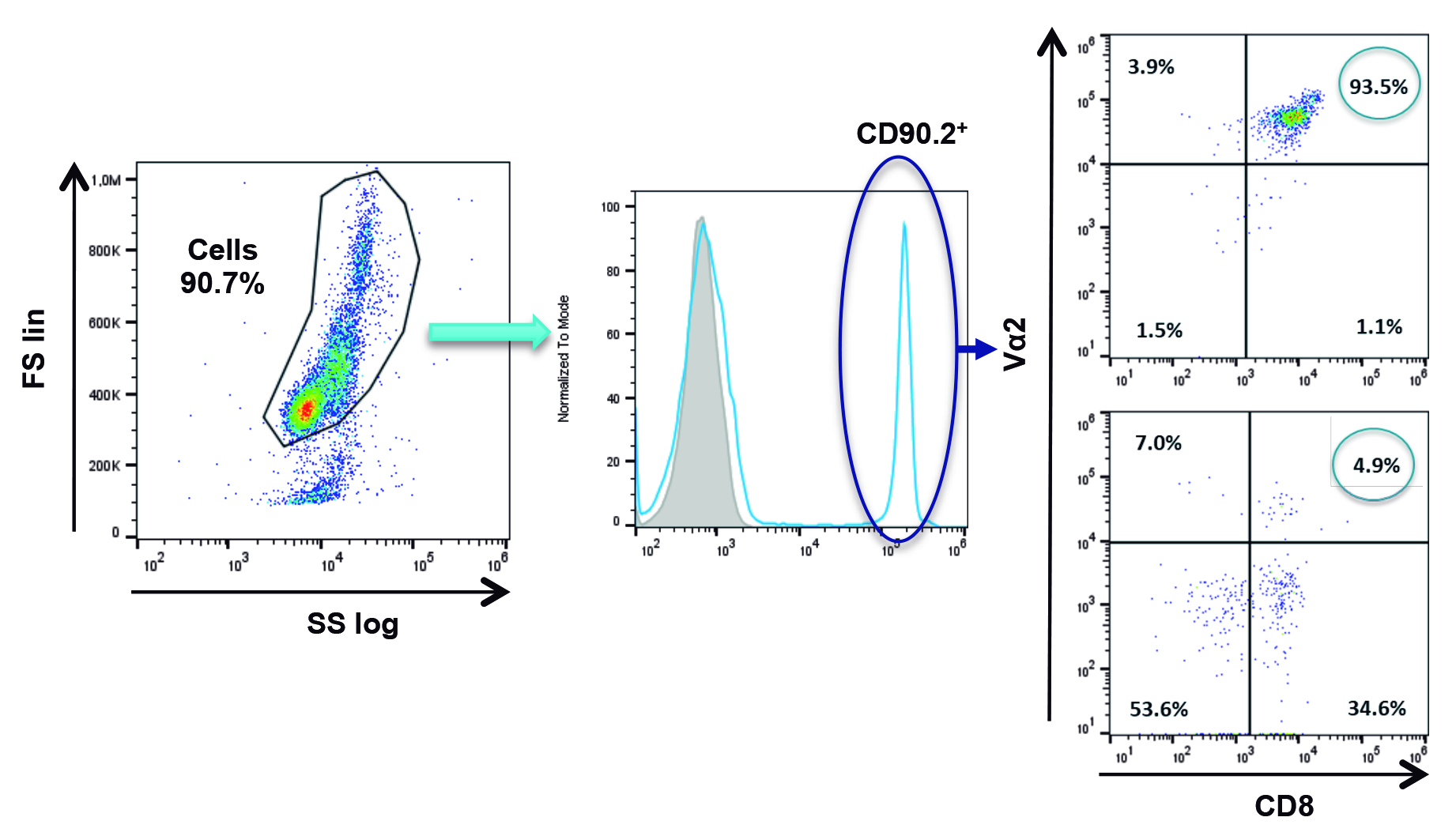
**

**Fig. S1. Phenotyping of blood obtained from the transgenic mouse strain OT-I.** Representative graphs, obtained using FlowJo software, showing presence of Vα2 in CD8^+^ cells within the population of mature T cells (CD90.2^+^). The upper panel shows the results obtained in a transgenic mouse while the lower panel displays the results in a mouse that has lost the transgene. Shaded grey histogram: isotype control; blue line: corresponding antibody.

**
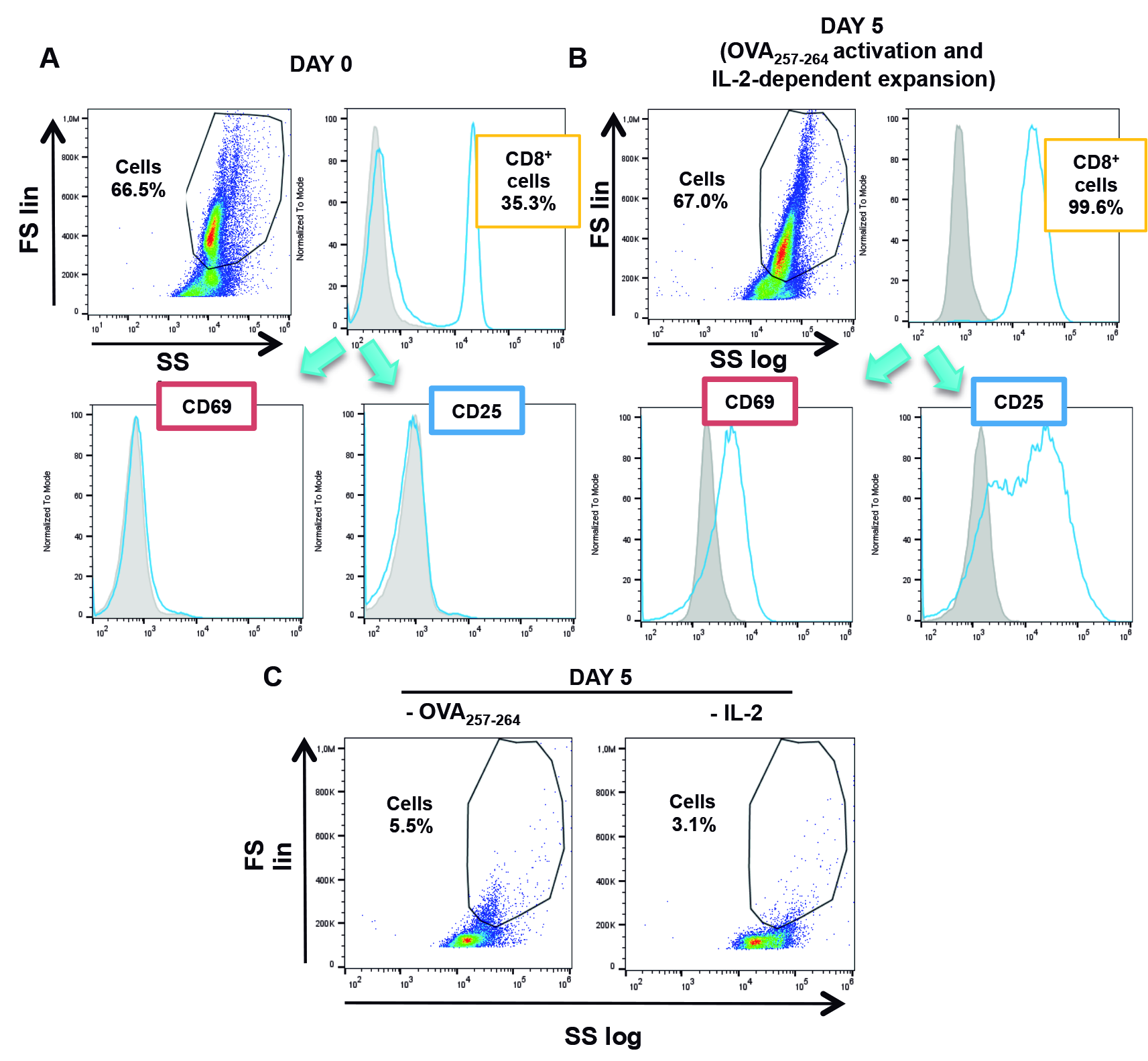
**

**Fig. S2. OT-I CD8^+^ T** **cell purification and expansion.** Representative graphs obtained using FlowJo software from the different populations at (A) day 0, after spleen and LN processing, and at (B) day 5, following activation with the OVA_257-264_ peptide and subsequent IL-2 expansion. The percentage of CD8^+^ cells and their activation profile were analysed using the surface markers CD69 and CD25. Shaded grey histogram: isotype control; blue line: corresponding antibody. (C) Representative graphs of the culture, at day 5, in the absence of the different stimuli (OVA_257-264_ or IL-2).

**
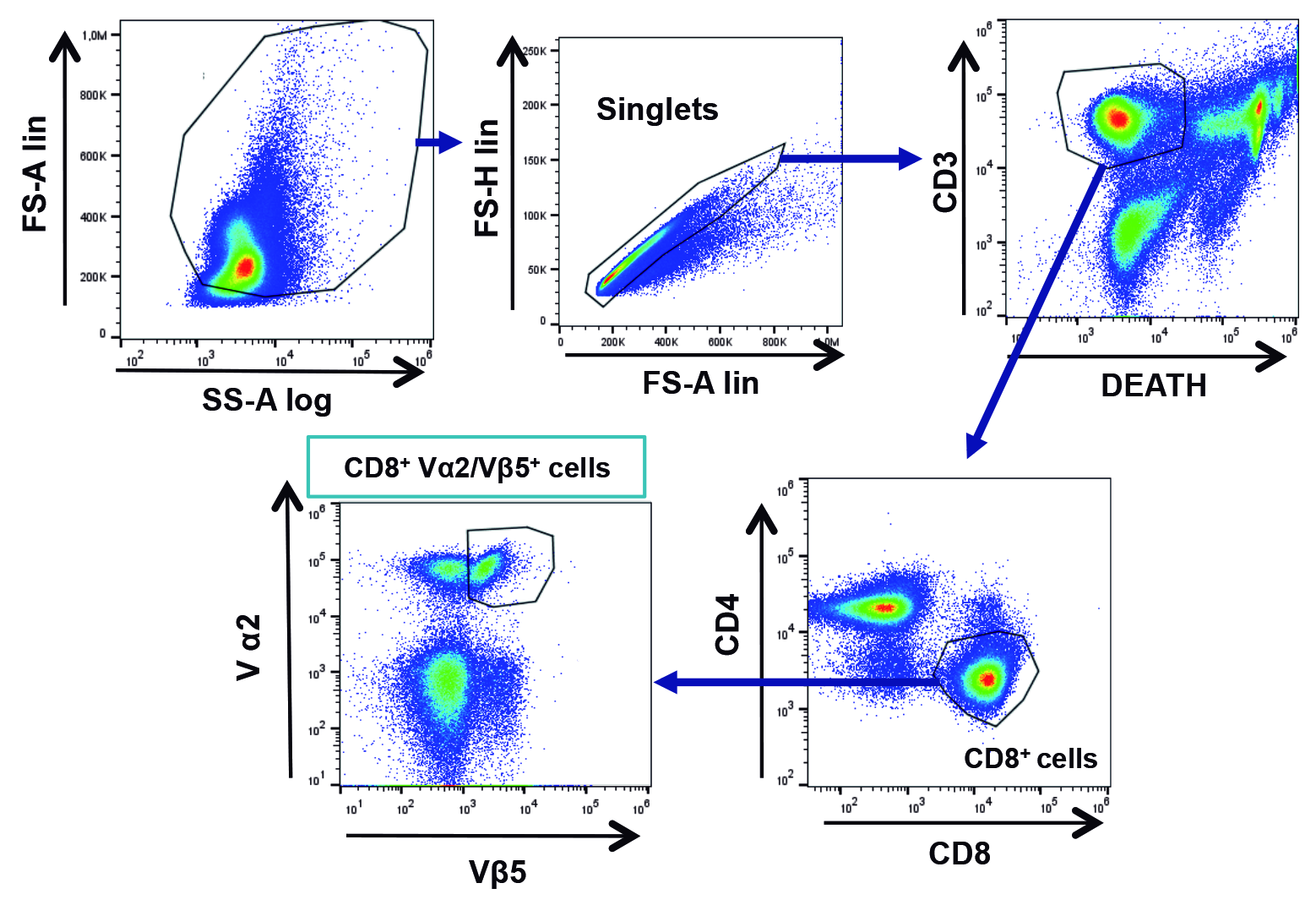
**

**Fig. S3. Analysis of CD8^+^Vα2/Vβ5^+^ T cell infiltration in the tumour-draining and distal LNs and in spleens.** Gating strategy followed for the analysis of the percentage of CD8^+^ Vα2 / Vβ5^+^ T cells present in the tumour-draining and distal LNs and in spleens.

**
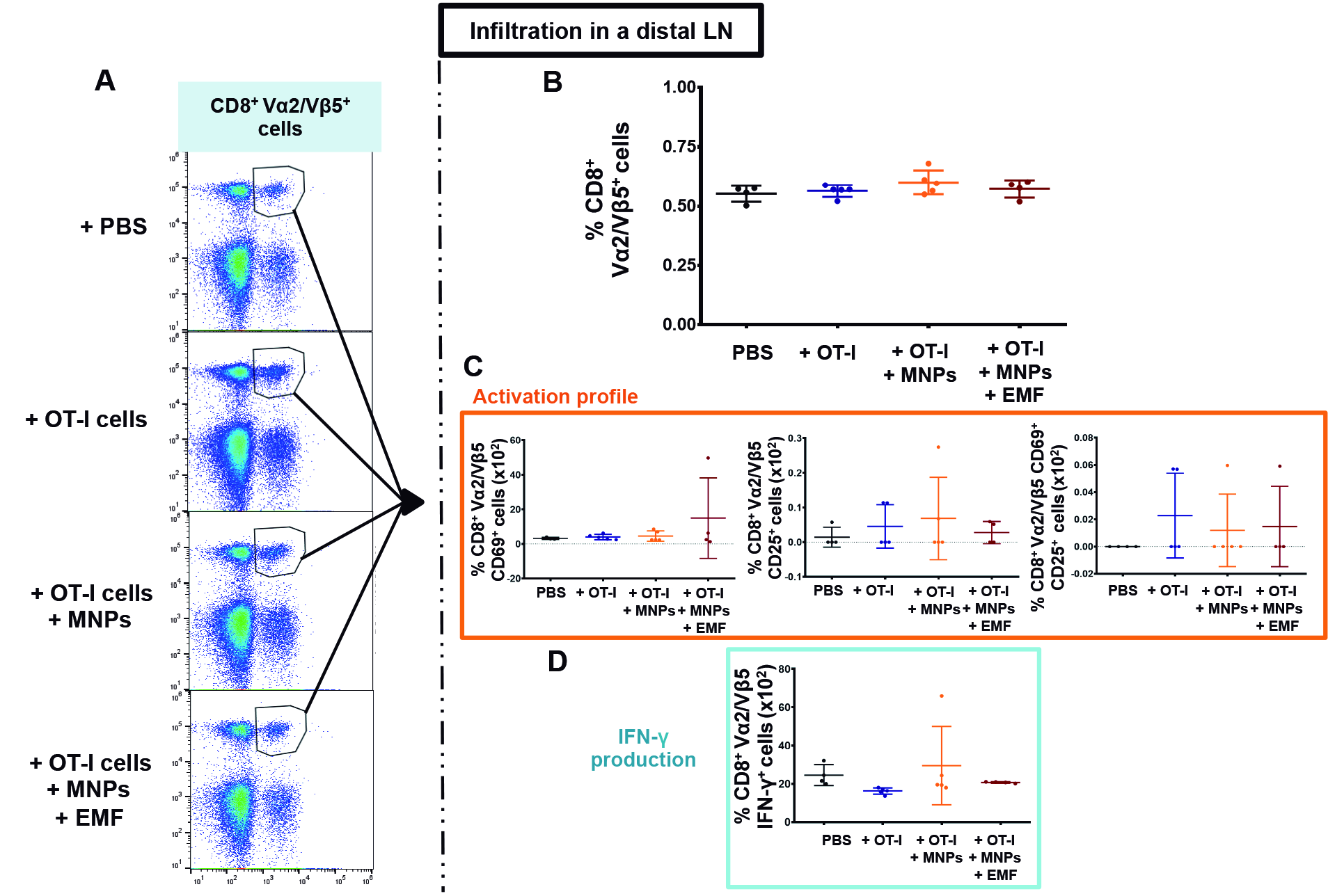
**

**Fig. S4. CD8^+^Vα2/Vβ5^+^ T cell infiltration of a distal LN in the different treatment groups**. (A) Representative dot plots showing the percentage of Vα2/Vβ5^+^ cells within the living CD3^+^CD8^+^ cell population. (B) Percentage of CD8^+^ Vα2/Vβ5^+^ T cells within living CD45^+^ cells in a distal LN for the different treatment groups. (C) Percentage of CD8^+^ Vα2/Vβ5^+^ CD69^+^, CD25^+^, and CD69^+^CD25^+^ T cells in a distal LN for the different treatment groups. (D) Percentage of CD8^+^ Vα2/Vβ5^+^ IFN-γ^+^ T cells in a distal LN for the different treatment groups. The results shown (mean ± SD) correspond to 4 or 5 mice / group, * p <0.05, ** p <0.01, *** p <0.001.

**
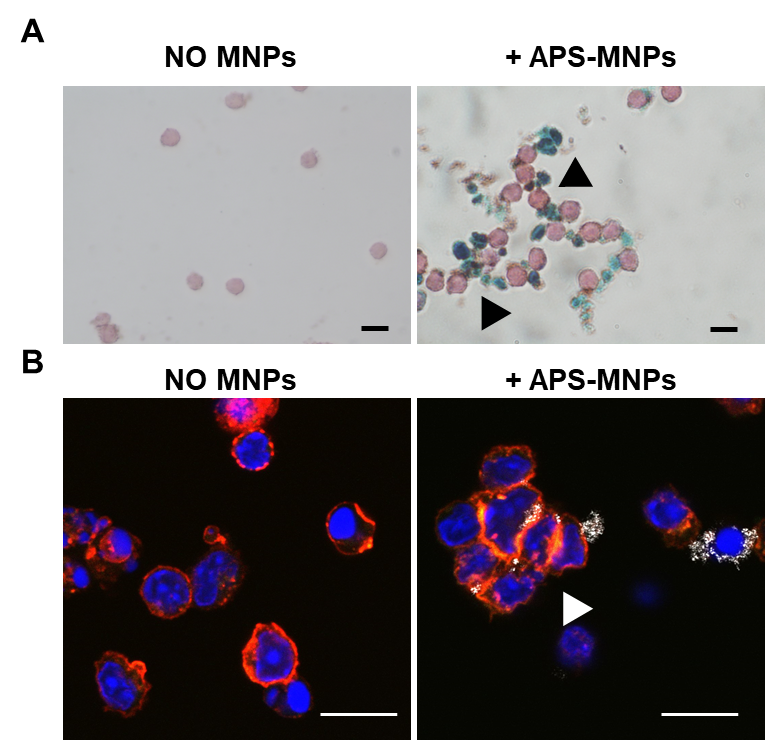
**

**Fig. S5. Cell aggregation after MNP treatment**. Representative images, showing cell aggregation due to MNP presence after 2 hour treatment, acquired using (A) Prussian blue staining and neutral red counterstain of OT-I CD8^+^ T cells after association with the MNPs (scale = 10 μm) or (B) confocal microscopy of the OT-I CD8^+^ T cells after treatment with APS-MNPs (cell membrane (red), MNPs (grey), and core (blue)) (scale = 10 μm). The arrows indicate the cell aggregation.

**
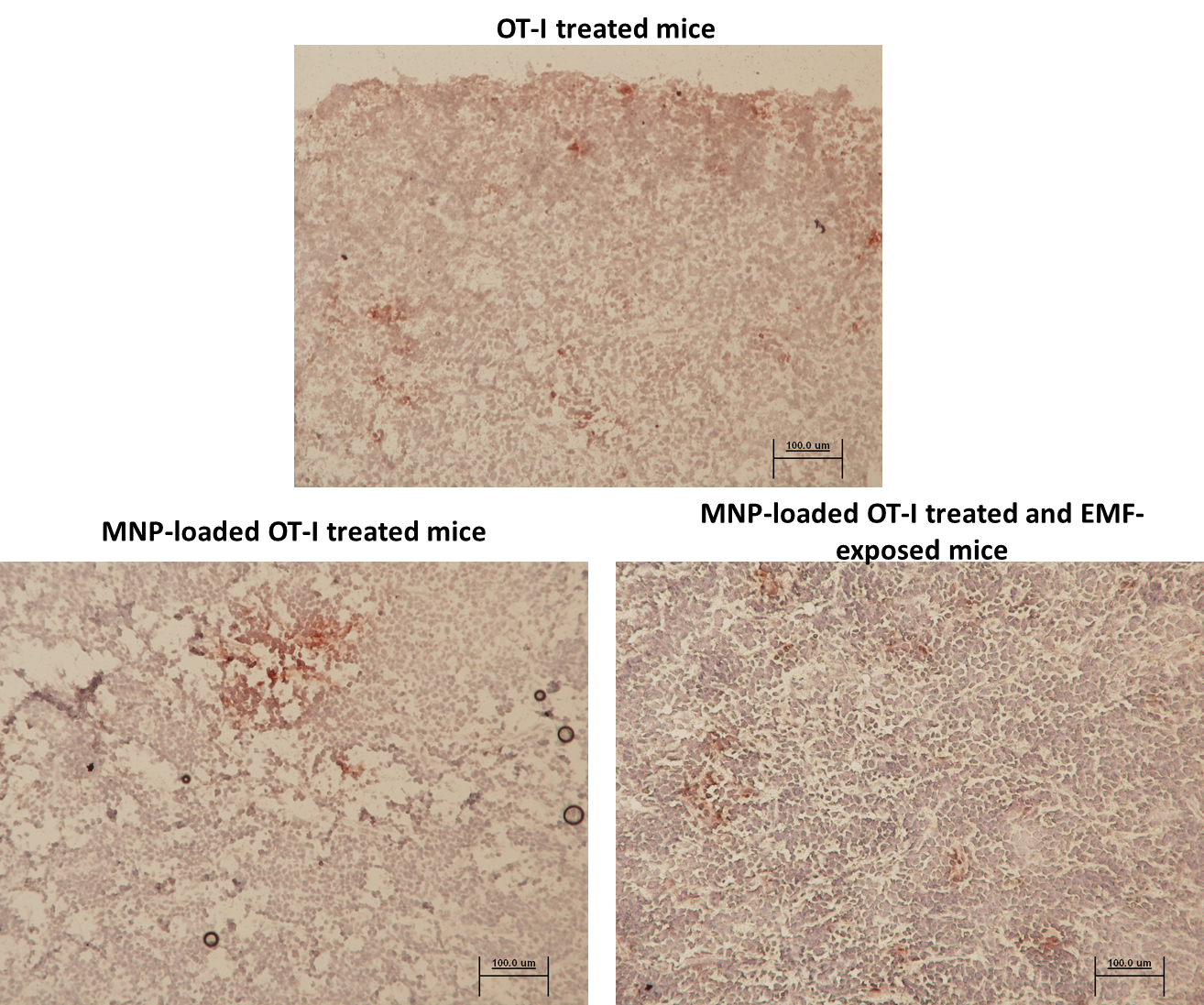
**

**Fig. S6. Immunohistochemical analysis of CD8^+^ T cell infiltration in tumours**. Immunohistochemical analysis of CD8^+^ T cell infiltration in the tumours from mice transferred with OT-I cells, MNP-loaded OT-I cells and MNP-loaded OT-I cells together with EMF-exposition. Representative images are shown (×10).

**SUPPLEMENTARY METHODS**

**Immunohistochemistry**

Tumours were removed and frozen in tissue-freezing medium (Jung). Immunohistochemical analyses were performed as previously described [1]. Briefly, sections were fixed, endogenous peroxidase was blocked, and slides incubated with anti-mouse CD8 primary antibody (YTS169.4, 1/100; abcam), followed by rat Histofine Simple Stain kits (Nichirei Biosciences), and finally stained with AEC+ substrate chromogen solution (Dako) and hematoxylin-counterstained.

[1] Spada R, Rojas JM, Pérez-Yagüe S, Mulens V, Cannata-Ortiz P, Bragado R and Barber DF: NKG2D ligand overexpression in lupus nephritis correlates with increased NK cell activity and differentiation in kidneys but not in the periphery. *J Leukoc Biol* 2015 Mar; 97(3): 583–598.

1. [↑](#footnote-ref-1)
